# Design of potent tyrosinase inhibiting *N*-arylated-4-yl-benzamides bearing 2-aminothiazole-triazole bi-heterocycles: mechanistic insight through enzyme inhibition, kinetics and computational studies†

**DOI:** 10.1039/d4ra01063a

**Published:** 2024-05-21

**Authors:** Farhan Mahmood Khan, Muhammad Athar Abbasi, Aziz-ur Rehman, Sabahat Zahra Siddiqui, Abdul Rehman Sadiq Butt, Hussain Raza, Mubashir Hassan, Syed Adnan Ali Shah, Muhammad Shahid, Song Ja Kim

**Affiliations:** a Department of Chemistry, Government College University Lahore 54000 Pakistan abbasi@gcu.edu.pk (+92)-42-111000010 Ext. 266; b Department of Biological Sciences, College of Natural Sciences, Kongju National University Gongju 32588 South Korea; c The Steve and Cindy Rasmussen Institute for Genomic Medicine, Nationwide Children Hospital Columbus Ohio 43205 USA; d Faculty of Pharmacy, Universiti Teknologi MARA Cawangan Selangor Kampus Puncak Alam Bandar Puncak Alam Selangor 42300 Malaysia; e Atta-ur-Rahman Institute for Natural Product Discovery (AuRIns), Universiti Teknologi MARA Cawangan Selangor Kampus Puncak Alam Bandar Puncak Alam Selangor 42300 Malaysia; f Department of Biochemistry, University of Agriculture Faisalabad 38040 Pakistan

## Abstract

By using a convergent methodology, a unique series of *N*-arylated 4-yl-benzamides containing a bi-heterocyclic thiazole-triazole core was synthesized and the structures of these hybrid molecules, 9a–k, were corroborated through spectral analyses. The *in vitro* studies of these multi-functional molecules demonstrated their potent mushroom tyrosinase inhibition relative to the standard used. The kinetics mechanism was exposed by lineweaver–burk plots which revealed that, 9c, inhibited mushroom tyrosinase non-competitively by forming an enzyme–inhibitor complex. The inhibition constant *K*_i_ calculated from Dixon plots for this compound was 0.016 μM. The computational study was also consistent with the experimental results and these molecules disclosed good results of all scoring functions and interactions, which suggested a good binding to mushroom tyrosinase. So, it was predicted from the inferred results that these molecules might be considered as promising medicinal scaffolds for the diseases associated with the over-expression of this enzyme.

## Abstract 2

By using a convergent methodology, a unique series of *N*-arylated 4-yl-benzamides containing a bi-heterocyclic thiazole-triazole core was synthesized and the structures of these hybrid molecules, 9a–k, were corroborated through spectral analyses.

## Graphical abstract

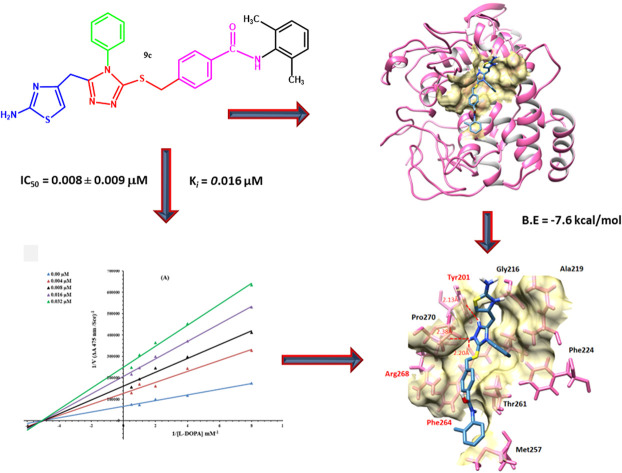

## 1. Introduction

Heterocyclic compounds are very important biologically and are found in nature as components of vitamins, alkaloids, hormones, and many other natural products. Several heterocycles are used in combinatorial and supramolecular chemistry, as well as in medicine, agriculture, and polymer industries.^1–3^

Thiazoles are a part of numerous natural products *e.g.*, epothilone, thiostrepton, thiamine pyrophosphate (TPP), carboxylase vitamin B_1_, and penicillin.^4,5^ Thiazoles are claimed to play a role in drug development for the treatment of allergies, HIV infections, hypertension, bacterial infections, schizophrenia and pain,^6^ as novel inhibitors of bacterial DNA gyrase B,^7^ and as fibrinogen receptor antagonists with antithrombotic activity.^8^ They displayed magnificent pharmaceutical activities, for instance, antifungal,^9^ antimicrobial,^10^ anti-inflammatory,^11^ analgesic,^12^ anticancer,^13^ and anticonvulsant activities.^14^

One of the isomeric forms of triazole is 1,2,4-triazole.^15^ These triazole compounds have various biological activities, including anticancer, antimicrobial, anti-inflammatory, antioxidant, anti-HIV and antimalarial.^16,17^ Some of the marketed drugs containing 1,2,4-triazole moiety, like anastrozole, letrozole, and vorozole are used for the treatment of breast cancer. Several compounds bearing 1,2,4-triazole moiety are stated as potent inhibitors of tyrosinase.^18^

Tyrosinase is a copper containing metalloenzyme with two Cu^+2^ ions in its active site. It is commonly found in plants, animals, and microorganisms.^19^ Tyrosinase is essential in facilitating the enzymatic browning of fruits and vegetables, which results in a significant loss of flavour and nutritional content.^20^ In humans, dopaquinone is converted to melanin by a series of reactions and tyrosinase plays an important role for its creation, which can result in hyperpigmentation disorders including melisma and seborrheic.^21^ Tyrosinase is an enzyme that is only present in melanoma cells, therefore, its blocking is helpful to develop a highly targeted treatment for skin cancer.^22^ However, it is probable that these inhibitors only target the cancer cells, leaving the DNA of healthy cells unaffected.^23–25^

Sulpiride and amisulpiride are two active benzamides which have been broadly applied in psychiatry and in the treatment of neurodegenerative disorders, schizophrenia, and Alzheimer's disease.^26,27^ Some benzamide derivatives have been reported to exhibit good enzyme inhibitory, antimicrobial, anti-inflammatory, serotonin (5-HT) and antitumor activities.^28,29^

**Fig. 1:**
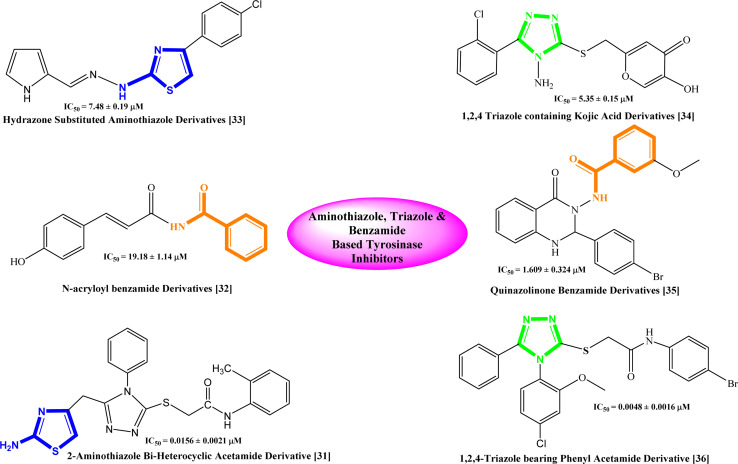
Rationale of the current study.

Based on the recent studies, phenolic compounds and their derivatives and several compounds including terpenoid, phenyl, pyridine, piperidine, pyridinone, hydroxypyridinone, thiosemicarbazone, thiosemicarbazide, azole, thiazolidine, kojic acid, benzaldehyde and xanthate derivatives were characterized as potent tyrosinase inhibitors.^30^ Similarly, some bi-heterocyclic compounds have been reported previously as tyrosinase inhibitors and some of them are shown with their IC_50_ value in Fig. 1,^31–36^ However, to completely cope with the issues of skin disorders, *i.e.* ephelides, melasma, and senile lentigines, it is imperative to find some more unique and secure inhibitors of this enzyme.

## 2. Results and discussion

### 2.1 Chemistry

**Scheme 1:**
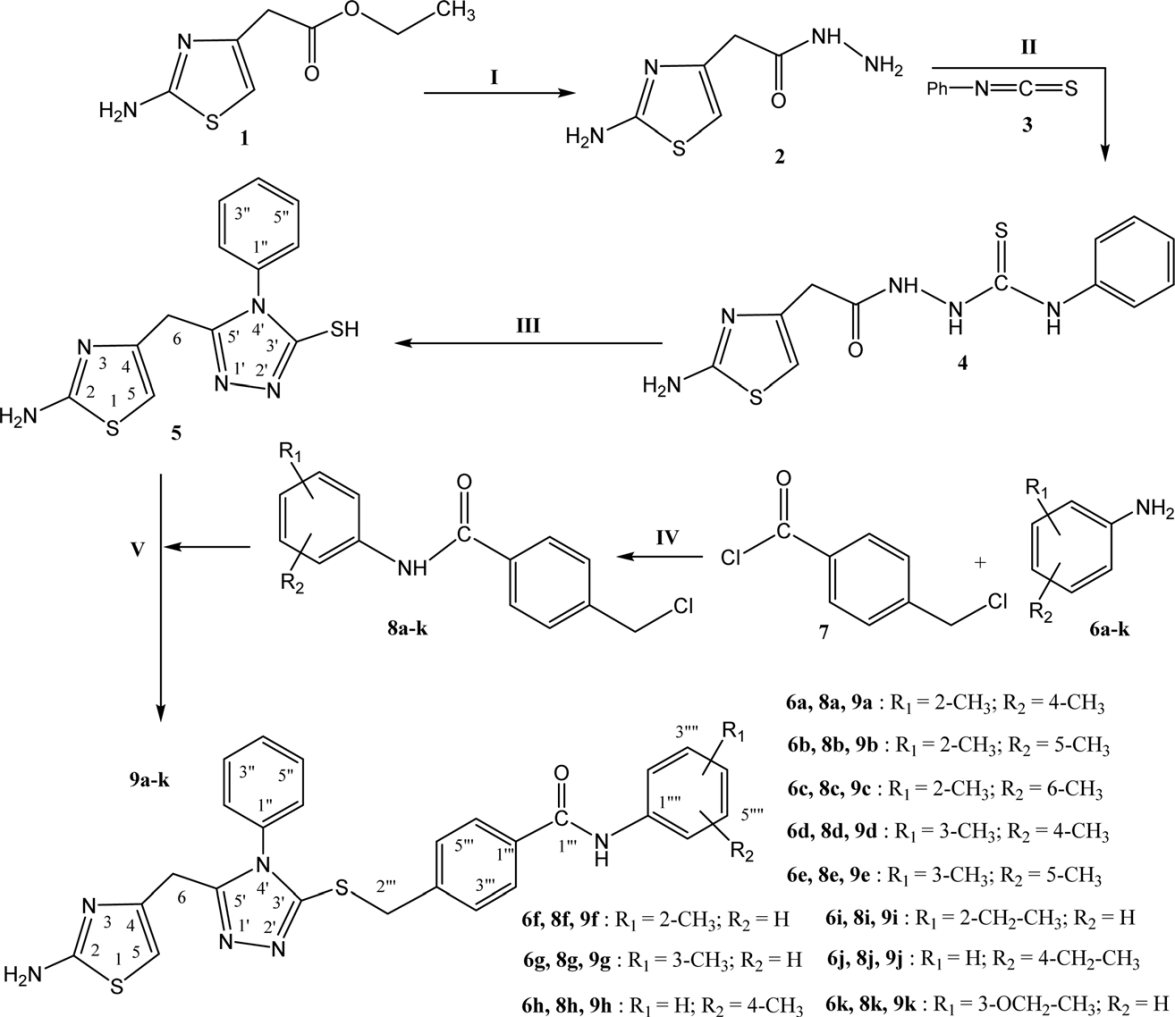
Outline for the synthesis of 4-[({5-[(2-amino-1,3-thiazol-4-yl)methyl]-4-phenyl-4*H*-1,2,4-triazol-3-yl}sulfanyl)methyl]-*N*-(aryl)benzamides. Reagents & conditions: (I) MeOH/N_2_H_4_·H_2_O/refluxing for 2 h (yield = 91%). (II) MeOH/3/refluxing for 1 h (yield = 88%). (III) The ppt. of 4 dissolved in 10% NaOH/filtration/acidification of filtrate in cold state to get ppt. of 5 (yield = 87%). (IV) Aqueos Na_2_CO_3_ solution/pH 9–10/vigorous manual shaking at RT for 2–3 h (yield = 89%). (V) DMF/LiH/stirring for 12–24 h (yield = 92–98%).

The designed bi-heterocyclic *N*-arylated benzamides were synthesized by a convergent protocol as described in Scheme 1. The synthesis was initiated by refluxing ethyl 2-(2-amino-1,3-thiazol-4-yl)acetate (1) in methanol and hydrazine hydrate to transform this starter into 2-(2-amino-1,3-thiazol-4-yl)acetohydrazide (2). The hydrazide, 2, was added in methanol and refluxed with phenyl isothiocyanate (3) to convert it into a solid intermediary compound 2-[2-(2-amino-1,3-thiazol-4-yl)acetyl]-*N*-phenyl-1-hydrazinecarbothioamide (4) which was cyclized to a nucleophilic molecule 5-[(2-amino-1,3-thiazol-4-yl)methyl]-4-phenyl-4*H*-1,2,4-triazole-3-thiol (5). In a complementary set of reactions, the substituted anilines (6a–k) were reacted with 4-chloromethyl benzoyl chloride (7) in an aqueous basic medium, to acquire the required electrophiles, 4-chloromethyl-*N*-(aryl)benzamides (8a–k). Finally, the nucleophile 5 was dissolved in DMF and a nip of LiH was added into it. To activate its mercapto position, the mixture was stirred for about 30 minutes, and in this last step, the electrophiles, 8a–k, were coupled in equimolar amounts with the activated 5 to yield the targeted bi-heterocyclic benzamides (9a–k).

**Fig. 2:**
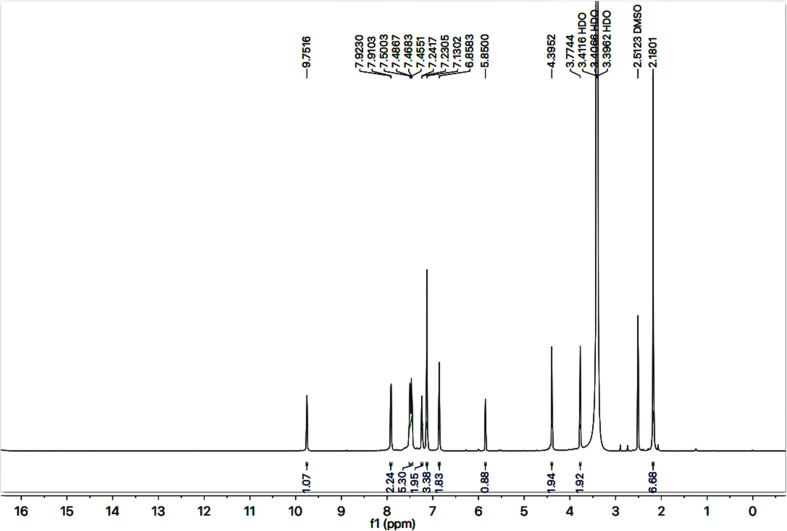
^1^H-NMR spectrum of compound 9c.

**Fig. 3:**
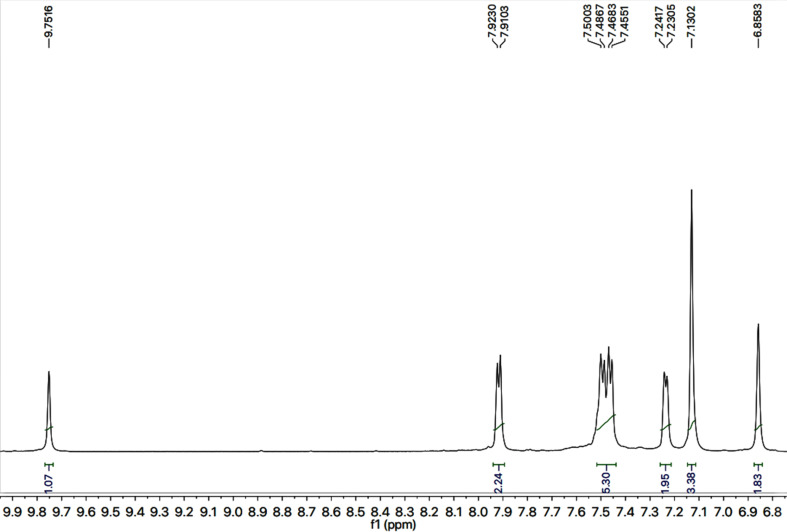
Down field region of ^1^H-NMR spectrum of 9c.

**Fig. 4:**
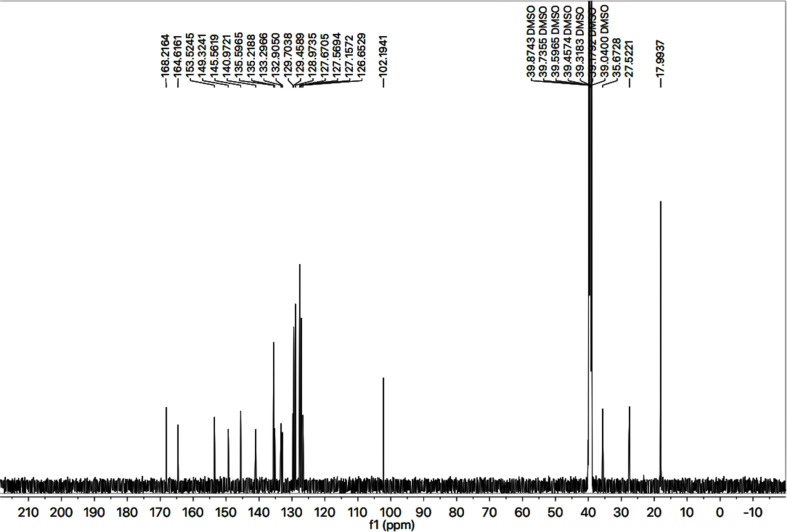
^13^C-NMR spectrum of compound 9c.

The detailed structural analysis of a compound, 9c, is discussed hereby. It was synthesized as a light brown amorphous solid, having melting point of 110–111 °C. The molecular formula, C_28_H_26_N_6_OS_2_, was affirmed by its CHN analysis data, supported by the count of number of protons in its ^1^H-NMR (Fig. 2 and 3) and carbons in its ^13^C-NMR spectrum (Fig. 4). Different functionalities in the molecule were ascertained by its IR spectrum. The characteristic peaks appeared at *ν* 3346 (N–H stretch), 3056 (C–H stretch), 2927 (–CH_2_– stretch), 1679 (C=O stretch), 1560 (C=C stretch), 1520 (C=N stretch), 1166 (C–N–C bond stretch), 626 (C–S stretch) cm^−1^. In its ^1^H-NMR spectrum, the phenyl ring linked with nitrogen (4′) of 1,2,4-triazole heterocyclic ring was identified by a broad doublet at 7.23 (br.d, *J* = 6.7, 2H, H-2′′ & H-6′′) and a multiplet in the aromatic region at *δ* 7.50–7.48 (m, 3H, H-3′′, H-4′′ & H-5′′). The 4-substituted benzamide unit was predictable by an amidic signal at *δ* 9.75 (s, 1H, –CO–NH-1′′′′) along with two prominent *ortho*-coupled broad doublets in the aromatic region at *δ* 7.91 (br. d, *J* = 7.6, 2H, H-2′′′ & H-6′′′) and 7.45 (br. d, *J* = 7.9, 2H, H-3′′′ & H-5′′′). The 2,6-dimethylphenyl moiety connected with nitrogen of benzamide unit was signified by a broad singlet at *δ* 7.13 (br.s, 3H, H-3′′′′, H-4′′′′ & H-5′′′′) in the aromatic region, along with a typical signal of two symmetrical methyl groups resonating at 2.18 (s, 6H, CH_3_-2′′′′ & CH_3_-6′′′′). The 2-amino-1,3-thiazol-4-yl unit was characterized by two singlets at *δ* 6.85 (br. s, 2H, H_2_N-2) and 5.85 (s, 1H, H-5), while a singlet at *δ* 3.77 (s, 2H, CH_2_-6) was assignable to a methylene group joining the two heterocycles in the molecule. Another singlet at *δ* 4.39 (s, 2H, CH_2_-8′′′) was rational for the methylene group linking the benzamide unit with the mercapto position of the triazole moiety.

**Fig. 5:**
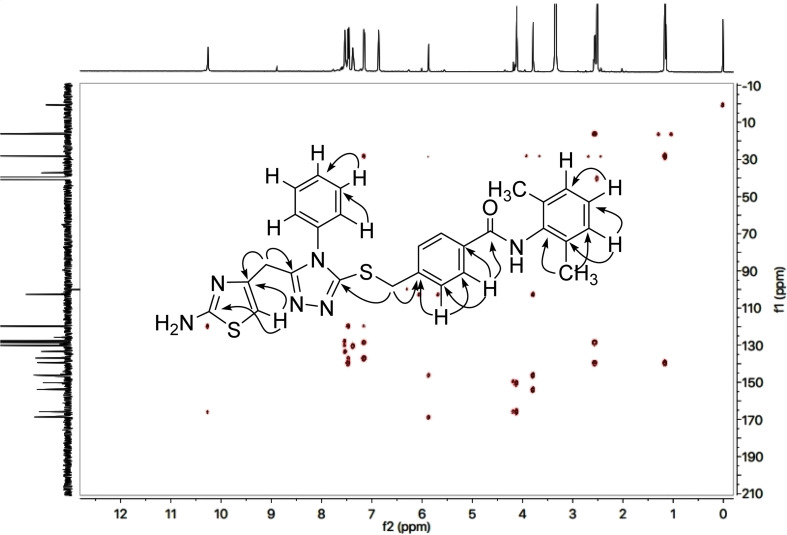
Important HMBC correlations of compound 9c.

The 2-amino-1,3-thiazol-4-yl moiety in its ^13^C-NMR spectrum (Fig. 4) was clearly indicated by two quaternary signals at *δ* 168.21 (C-2), and 145.56 (C-4), along with a methine signal at *δ* 102.19 (C-5). Similarly, the other heterocycle *i.e.* (1,2,4-triazloe-2-yl)sulfanyl was also identified by two quaternary signals at *δ* 153.52 (C-5′) and 149.32 (C-3′) while a methylene linking the two heterocycles (4-position of the former heterocycle with 5′-position of the latter heterocycle) was apparent at *δ* 27.52 (C-6). The 2,6-dimethylphenyl moiety was also corroborated with two quaternary signals at *δ* 135.59 (C-2′′′′ & C-6′′′′) and 135.21 (C-1′′′′), along with symmetrical duplet methines at *δ* 127.56 (C-3′′′′ & C-5′′′′) and another methine at *δ* 126.65 (C-4′′′′). The symmetrical 2,6-dimethyl groups on this aromatic ring were verified by a signal at *δ* 17.99 (CH_3_-2′′′′ & CH_3_-6′′′′). The 4-substituted benzamide unit was established by a distinct signal of carbonyl carbon at *δ* 164.61 (C-7′′′) in addition to other two quaternary signals at *δ* 140.97 (C-4′′′), and 133.29 (C-1′′′) along with two methine duplet resonances at *δ* 128.97 (C-3′′′ & C-5′′′) and 127.67 (C-2′′′ & C-6′′′). The signal at *δ* 35.67 (C-8′′′) was assignable to a methylene joining the benzamide from its 4-position to the sulfur atom joined with 1,2,4-triazole heterocycle. The phenyl group attached to the nitrogen (4′) atom of 1,2,4-triazole ring was distinctive by a quaternary signal at *δ* 132.90 (C-1′′), a methine signal at 129.70 (C-4′′) along with two symmetrical methine duplets at *δ* 129.45 (C-3′′ & C-5′′) and *δ* 127.15 (C-2′′ & C-6′′). The C–H connectivities in the carbon skeleton were comprehensively certified by its HMBC spectrum and the important correlationsare demonstrated on this spectrum in (Fig. 5).

**Fig. 6:**
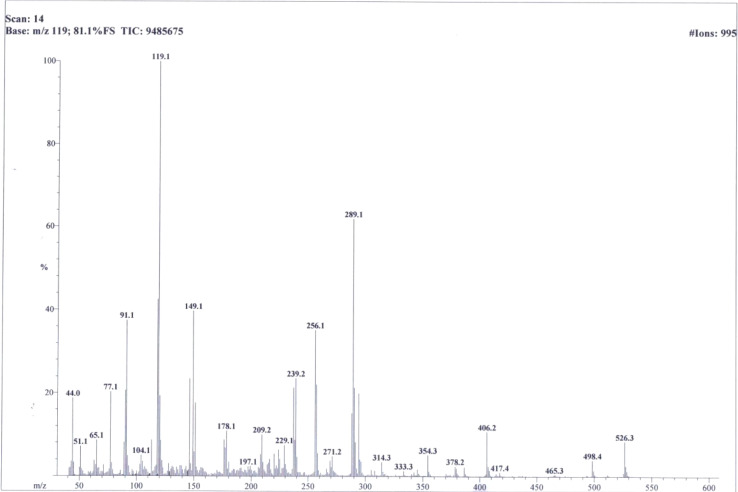
EI-MS spectrum of compound 9c.

So, based upon aforementioned evidences and the EIMS spectra of compound 9c (Fig. 6) the structure of 9c was confirmed and it was named as 4-[({5-[(2-amino-1,3-thiazol-4-yl)methyl]-4-phenyl-4*H*-1,2,4-triazol-3-yl}sulfanyl)methyl]-*N*-(2,6-dimethylphenyl)benzamide. Similarly, the structures of all other synthesized derivatives were verified through rigorous spectral analyses (Fig. S1–S20†).

### 2.2 Mushroom tyrosinase inhibition

**Fig. 7:**
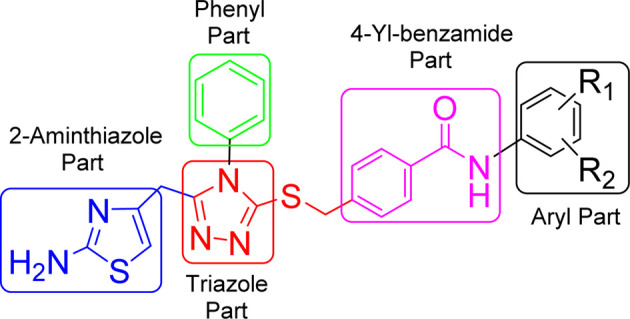
General structural features of compounds 9a–k.

These synthesized bi-heterocyclic benzamides (9a–k) were assessed for their inhibiting potentials against tyrosinase enzyme and their results are tabulated in Table 2. All these compounds exhibited very potent inhibitory activities against this enzyme, as evident from their lower IC_50_ (μM) values as compared to the standard, kojic acid, having IC_50_ value of 16.8320 ± 1.1600 μM. Though the rendered activity is an accumulative attribute of a whole molecule, yet a limited structure–activity relationship (SAR) was recognized by examining the effect of different aryl entities on the inhibitory potential, as it was the only varying part in all these molecules. The general structural parts of the studied benzamides are featured in Fig. 7.

### 2.3 Structure–activity relationship

**Fig. 8:**
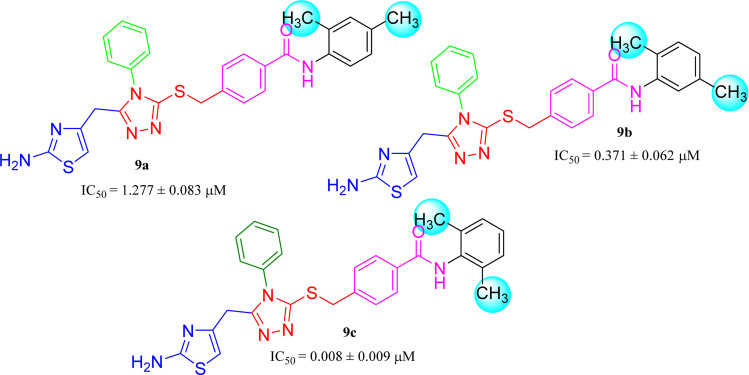
SAR of compounds 9a, 9b, and 9c.

Amongst three di-methylated regio-isomers, compound 9c in which the methyl groups were present at both *ortho* positions exhibited better inhibitory activity (IC_50_ = 0.008 ± 0.009 μM) as compared to other isomers in which methyl groups were on different positions (2,4-dimethylphenyl, 2,5-dimethylphenyl). These analogues 9a and 9b possessed comparatively high IC_50_ values; 1.277 ± 0.083 and 0.371 ± 0.062 μM, indicating that the presence of the methyl groups at symmetrical *ortho*-positions in former molecule, probably increased its appropriate interactions with the active site of the enzyme (Fig. 8).

**Fig. 9:**
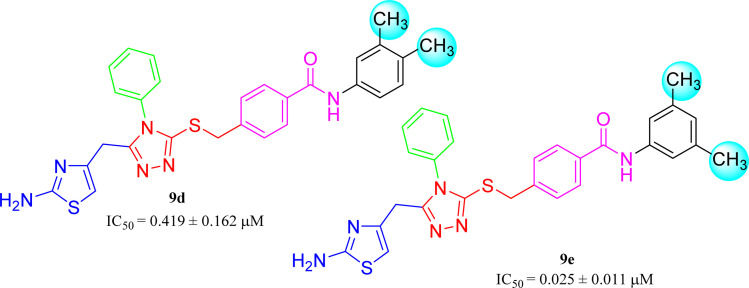
SAR of compounds 9d and 9e.

Similarly, when the inhibitory potential of other two di-methylated isomers was compared in which the one small-sized methyl group was fixed at 3-postion, it was observed that 9d with adjacent methyl groups (3,4-dimethylphenyl), had a lesser activity (IC_50_ = 0.419 ± 0.162 μM), as compared to 9e (IC_50_ = 0.025 ± 0.011 μM). Indeed, the molecule 9e was the second most potent among the series bearing the methyl groups now at *meta* but again the symmetrical positions in aryl part, which is presumed to augment the appropriate interactions with the active site of the enzyme and increase its inhibitory activity (Fig. 9).

**Fig. 10:**
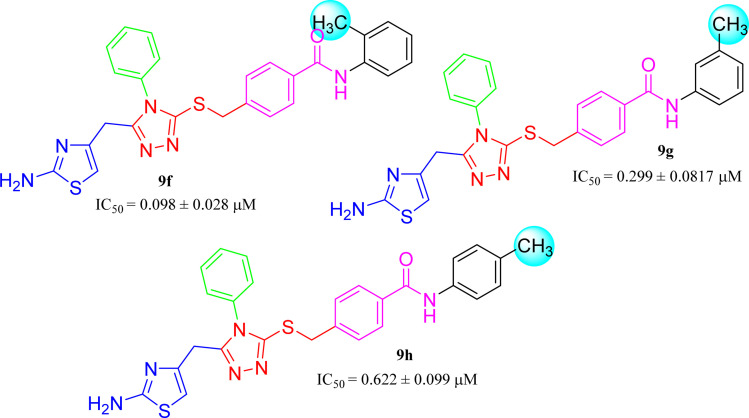
SAR of compounds 9f, 9g and 9h.

The three *mono*-methylated compounds 9f, 9g and 9h having IC_50_ = 0.098 ± 0.028, 0.299 ± 0.081 and 0.622 ± 0.099 μM, showed variable activities owing to change in the position of the methyl group in the aryl part. The molecule 9f in which the methyl group was at *ortho*-position and closer to the benzamido group displayed superb activity while a decreasing trend was observed when the methyl group was present away from the benzamido group, in *meta* and *para*-isomer (Fig. 10).

**Fig. 11:**
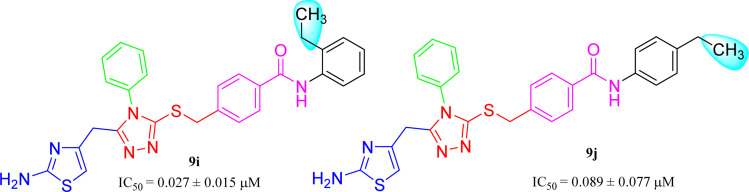
SAR of compounds 9i and 9j.

The compound 9i has a non-polar ethyl group at the *ortho*-position while 9j has the same ethyl group at *para*-position (Fig. 11). A similar trend was observed in these *mono*-substituted molecules, as the *ortho*-isomer (IC_50_ = 0.027 ± 0.015 μM), probably due to a compactness in size, possessed better inhibitory activity as compared to the respective *para*-isomer (IC_50_ = 0.089 ± 0.077 μM). Anyhow, these two compounds displayed impressive inhibition relative to *mono*-methylated molecules, presented in the aforementioned set. This order was also consistent to some extent with the +I observations, as the ethyl group has more +I effect than methyl group.

**Fig. 12:**
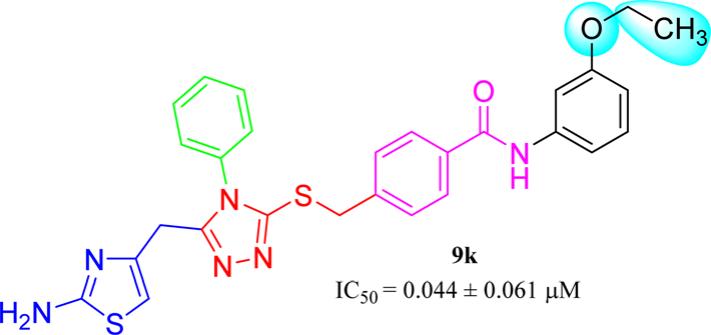
SAR of compound 9k.

9k (IC_50_ = 0.044 ± 0.061 μM) has medium-sized polar ethoxy group at *meta* position. Owing to varied nature of this group, having −I, and +R effects, it was presumed to exhibit good interactions with the enzyme and the molecule overall exhibited a considerably good inhibitory potential (Fig. 12).

### 2.4 Kinetic analysis

**Fig. 13:**
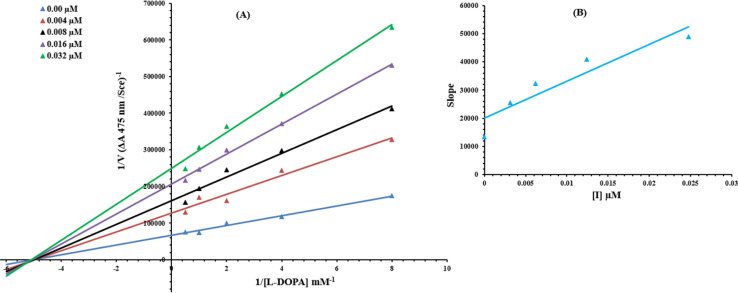
Lineweaver–Burk plots for inhibition of tyrosinase in the presence of compound 9c. (A) Concentrations of 9c were 0.00, 0.004, 0.008, 0.016 and 0.032 μM, respectively. Substrate l-DOPA concentrations were 0.125, 0.25, 0.5, 1 and 2 mM, respectively. (B) The insets represent the plot of the slope *versus* inhibitor 9c concentrations to determine inhibition constant. The lines were drawn using linear least squares fit.

To understand the inhibitory mechanism of synthetic compounds on the tyrosinase inhibition, kinetic study was performed. Based on our IC_50_ results, the most potent compound 9c was selected to determine their inhibition type and inhibition constant. The kinetic results of the enzyme by the Lineweaver–Burk plot of 1/*V versus* 1/[*S*] in the presence of different inhibitor concentrations furnished a series of straight lines (Fig. 13A). The results of 9c showed that compound intersected within the second quadrant. The analysis showed that *V*_max_ decreased to new increasing doses of inhibitors on the other hand *K*_m_ remained the same. This behavior indicated that 9c inhibited the tyrosinase non-competitively to form the enzyme–inhibitor complex. The secondary plot of slope against the concentration of inhibitors showed enzyme inhibitor dissociation constant (*K*_i_) (Fig. 13B).

### 2.5 Hemolytic activity

The synthesized compounds, 9a–k, were also exposed to hemolytic assay to ascertain their harmless utility as medicinal scaffolds. Cytotoxicity was profiled as percentage of hemolysis and the results are shown in Table 1. It was obvious from the results that all the derivatives of this series had modest toxicity towards red blood cell membrane as compared to the positive control, Triton X (94.11 ± 0.01%). The minimum toxicity was rendered by 9h (2.64 ± 0.04%) in the series. In general, it was rational that most of the molecules exhibited very moderate cytotoxicity and thus can be deliberated as safe therapeutic agents for further applications in drug designing program.

**Table 1:** Tyrosinase inhibitory and hemolytic activity of bi-heterocyclic benzamides, 9a–k^a^^b^

Compounds	Aryl part	Mushroom tyrosinase activity IC_50_ ± SEM (μM)	Hemolysis (%) (mean ± SEM)
9a	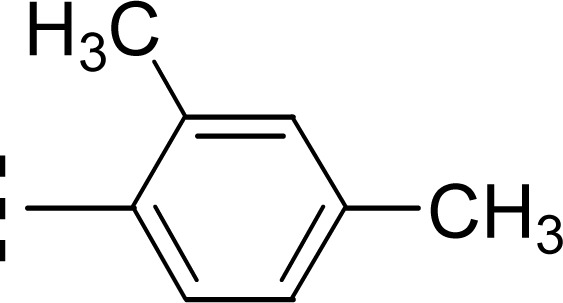	1.277 ± 0.083	6.47 ± 0.02
9b	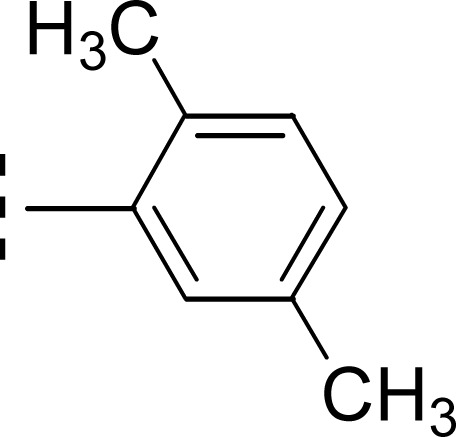	0.371 ± 0.062	11.76 ± 0.03
9c	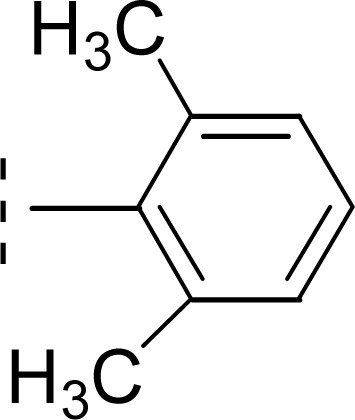	0.008 ± 0.009	9.41 ± 0.02
9d	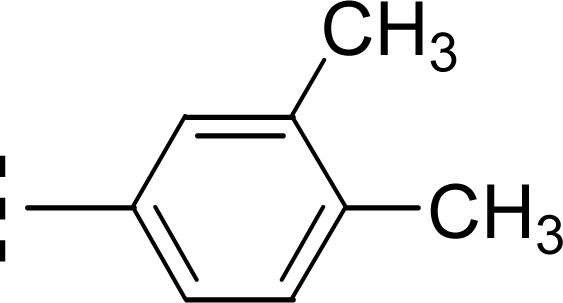	0.419 ± 0.162	8.82 ± 0.04
9e	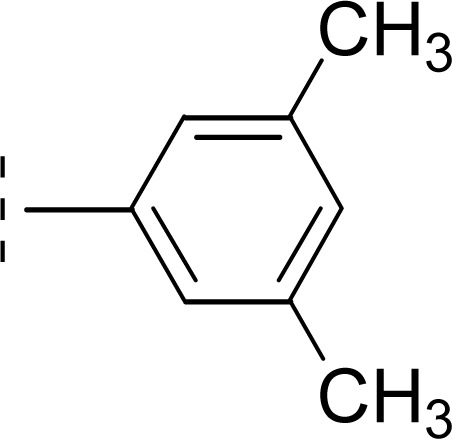	0.025 ± 0.011	6.47 ± 0.02
9f	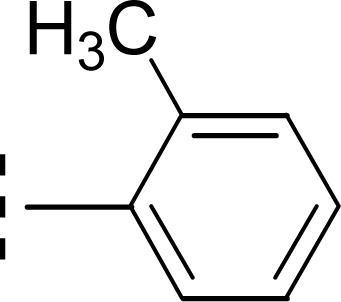	0.098 ± 0.028	12.94 ± 0.03
9g	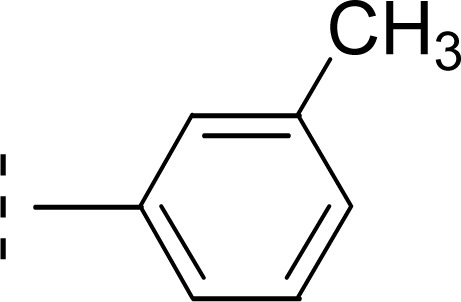	0.299 ± 0.081	6.52 ± 0.02
9h	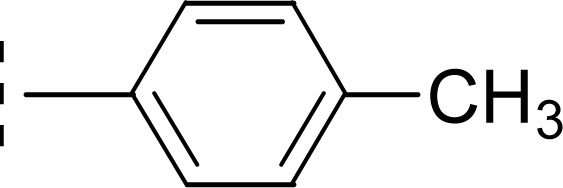	0.622 ± 0.099	2.64 ± 0.04
9i	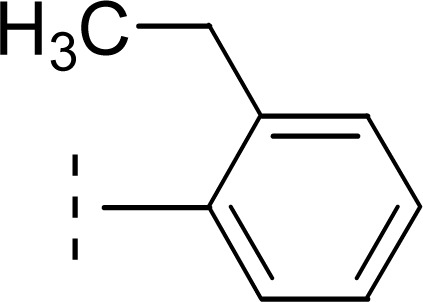	0.027 ± 0.015	9.41 ± 0.05
9j	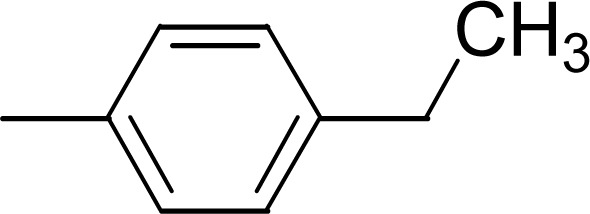	0.089 ± 0.077	8.52 ± 0.05
9k	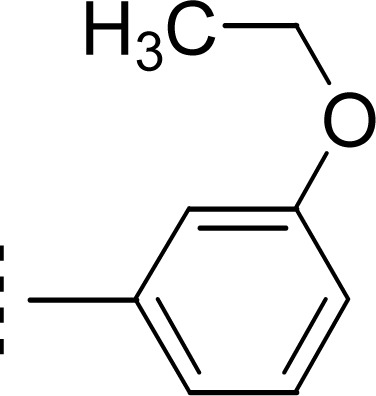	0.044 ± 0.061	10.35 ± 0.03
Kojic acid		16.832 ± 1.162	—
Triton X		—	94.11 ± 0.01

a SEM = standard error of the mean; values are expressed in mean ± SEM.

b PBS hemolysis = 0.00 ± 0.01%.

### 2.6 Docking studies of 9a–k into mushroom tyrosinase enzyme

The docked complexes of all the compounds 9a–k against mushroom tyrosinase were analyzed separately and evaluated because of minimum energy values (kcal mol^−1^) and ligand binding interactions (hydrogen/hydrophobic) pattern. Results showed that all compounds showed good binding energy values while binding to the target protein (Table 2). Prior research showed that the standard error for Autodock is testified as 2.5 kcal mol^−1^. However, in all docking complexes the predicted energy values difference was less than standard energy value. Although, the basic nucleus of all the synthesized compounds was similar, therefore most of ligands possess good efficient energy values and have no large energy fluctuations difference.

**Table 2:** Docking energy values of docked complexes

Ligands-protein docking complexes	Binding affinity (kcal mol^−1^)
9a	−6.9
9b	−8
9c	−7.6
9d	−6.2
9e	−6.6
9f	−8.3
9g	−6.5
9h	−6.6
9i	−8.5
9j	−7.1
9k	−6.8

#### 2.6.1 Binding analyses of synthesized compounds against mushroom tyrosinase

**Fig. 14:**
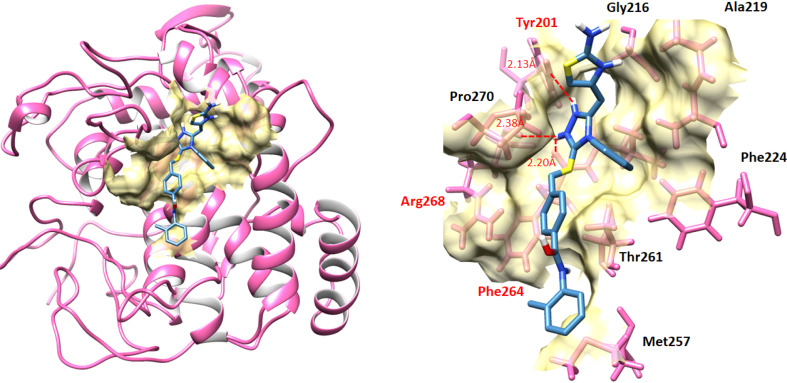
Binding interaction pattern between 9c and target protein.

The ligands-protein binding analyses showed that 9c confined in the active binding pocket of target protein as mentioned in Fig. 14. The docking result of 9c-receptor docked complex showed that hydrogen bonds were observed at Asn-260 residue. The hydrogen atom of 9c form hydrogen bond against Asn-260 with bond length 2.78 Å. Our docking results showed good correlation with published research which strengthens our work and efficacy. The 2D conformations and binding pose and interactions with binding residues of all the candidate molecules are mentioned in (Fig. S21–S30†).

## 3. Experimental

### 3.1 General

All the reagents were purchased from Sigma Aldrich and Alfa Aesar (Germany). Analytical grade solvents were delivered by local suppliers. Melting points were measured on Griffin and George apparatus by using capillary tubes and were found uncorrected. Initial purity of compound was documented by pre-coated silica gel aluminum plates in thin layer chromatography (TLC). Ethyl acetate and *n*-hexane were used as solvent system in ratio (30 : 70). The spots were recognized by UV_254_. IR peaks were documented on a Jasco-320-A spectrometer by using KBr pellet method. By using BBO probe in Bruker Advance III 600 As-cend spectrometer, signals were recorded in DMSO-d_6_ of ^1^H-NMR (*δ*, ppm) at 600 MHz and ^13^C-NMR at 150 MHz. Foss Heraeus CHN–O-rapid instrument was used for elemental analyses and theoretical values were within ±0.4%. Spectra of EI-MS was obtained by JEOL JMS-600H instrument with data processing system. Value of chemical shift (*δ*) is given in ppm and coupling constant (*J*) in Hz.

### 3.2 Synthesis of 5-[(2-amino-1,3-thiazol-4-yl)methyl]-4-phenyl-4*H*-1,2,4-triazole-3-thiol (5)

Synthesis of 5-[(2-amino-1,3-thiazol-4-yl)methyl]-4-phenyl-4*H*-1,2,4-triazole-3-thiol (5). Starting from ethyl 2-(2-amino-1,3-thiazol-4-yl)acetate (1), the compound 5 was synthesized by following the reported protocols by our group.^37^

### 3.3 General synthesis of 4-[({5-[(2-amino-1,3-thiazol-4-yl)methyl]-4-phenyl-4*H*-1,2,4-triazol-3-yl}sulfanyl)methyl]-*N*-(aryl)benzamides (9a–k)

*N*,*N*-Dimethyl formamide (DMF, 3 mL) was taken in 100 mL round bottom flask and 5-[(2-amino-1,3-thiazol-4-yl)methyl]-4-phenyl-4*H*-1,2,4-triazole-3-thiol (0.2 g, 5) was dissolved in it. Added one pinch of LiH in this solution and mixture was stirred for half an hour at room temperature. After that equimolar amount of different electrophiles, *N*-aryl-4-chlorobenzamide (8a–k, one in each reaction), were added in each respective reaction and stirred for 12–24 hours. The completion of reaction was monitored by TLC. The *n*-hexane and ethyl acetate were used to make a solvent system in the ratio of 70 : 30. Single spot of product was the sign of completion of reaction. The product was precipitated out by adding excess ice-cold distilled water. The targeted products, 9a–k, were obtained through filtration, excess washing with distilled water and dried respectively for further use.

#### 3.3.1 4-[({5-[(2-Amino-1,3-thiazol-4-yl)methyl]-4-phenyl-4*H*-1,2,4-triazol-3-yl}sulfanyl)methyl]-*N*-(2,4-dimethylphenyl)benzamide (9a)

Mol. formula: C_28_H_26_N_6_OS_2_; mol. mass: 526 g mol^−1^; IR (KBr, *ν*/cm^−1^): 3346 (N–H str.), 3052 (C–H str. of aromatic ring), 2920 (–CH_2_– str.), 1670 (C=O str.), 1562 (C=C str. of aromatic ring), 1519 (C=N str.), 1167 (C–N–C bond str.), 621 (C–S str.); ^1^H-NMR (600 MHz, DMSO-d_6_, *δ*/ppm): 9.77 (s, 1H, –CO–NH-1′′′′), 7.88 (br.d, *J* = 7.8, 2H, H-2′′′ & H-6′′′), 7.52–7.47 (m, 3H, H-3′′, H-4′′ & H-5′′), 7.43 (br.d, *J* = 7.9, 2H, H-3′′′ & H-5′′′), 7.22 (br.d, *J* = 7.4, 2H, H-2′′ & H-6′′), 7.17 (br.d, *J* = 7.81, 1H, H-6′′′′), 7.07 (br.s, 1H, H-3′′′′), 7.01 (br.d, *J* = 7.8 1H, H-5′′′′), 6.85 (br.s, 2H, H_2_N-2), 5.83 (s, 1H, H-5), 4.38 (s, 2H, CH_2_-8′′′), 3.76 (s, 2H, CH_2_-6), 2.28 (s, 3H, CH_3_-4′′′′), 2.17 (s, 3H, CH_3_-2′′′′); ^13^C-NMR (150 MHz, DMSO-d_6_, *δ*/ppm): 168.21 (C-2), 164.88 (C-7′′′), 153.50 (C-5′), 149.31 (C-3′), 145.57 (C-4), 140.88 (C-4′′′), 135.05 (C-1′′′′), 133.70 (C-1′′′), 133.52 (C-2′′′′), 133.51 (C-4′′′′), 132.91 (C-1′′), 130.78 (C-5′′′′), 129.70 (C-4′′), 129.47 (C-3′′ & C-5′′), 128.91 (C-3′′′ & C-5′′′), 127.65 (C-2′′′ & C-6′′′), 127.16 (C-2′′ & C-6′′), 126.52 (C-3′′′′), 126.46 (C-6′′′′), 102.19 (C-5), 35.67 (C-8′′′), 27.52 (C-6), 20.51 (CH_3_-4′′′′), 17.76 (CH_3_-2′′′′). Anal. calc. for C_28_H_26_N_6_OS_2_ (526.67): C, 63.85; H, 4.98; N, 15.96. Found: C, 63.81; H, 4.93; N, 15.92.

#### 3.3.2 4-[({5-[(2-Amino-1,3-thiazol-4-yl)methyl]-4-phenyl-4*H*-1,2,4-triazol-3-yl}sulfanyl)methyl]-*N*-(2,5-dimethylphenyl)benzamide (9b)

Mol. formula: C_28_H_26_N_6_OS_2_; mol. mass.: 526 g mol^−1^; IR (KBr, *ν*/cm^−1^): 3353 (N–H str.), 3050 (C–H str. of aromatic ring), 2926 (–CH_2_– str.), 1676 (C=O str.), 1569 (C=C str. of aromatic ring), 1527 (C=N str.), 1165 (C–N–C bond str.), 633 (C–S str.); ^1^H-NMR (600 MHz, DMSO-d_6_, *δ*/ppm): 9.80 (s, 1H, –CO–NH-1′′′′), 7.87 (br.d, *J* = 7.8, 2H, H-2′′′ & H-6′′′), 7.51–7.47 (m, 3H, H-3′′, H-4′′ & H-5′′), 7.44 (br.d, *J* = 7.9, 2H, H-3′′′ & H-5′′′), 7.22 (br.d, *J* = 7.0, 2H, H-2′′ & H-6′′), 7.15–7.14 (m, 2H, H-3′′′′ & H-6′′′′), 6.98 (br.d, *J* = 7.5, 1H, H-4′′′′), 6.85 (br.s, 2H, H_2_N-2), 5.84 (s, 1H, H-5), 4.39 (s, 2H, CH_2_-8′′′), 3.77 (s, 2H, CH_2_-6), 2.28 (s, 3H, CH_3_-5′′′′), 2.17 (s, 3H, CH_3_-2′′′′); ^13^C-NMR (150 MHz, DMSO-d_6_, *δ*/ppm): 168.22 (C-2), 164.90 (C-7′′′), 153.52 (C-5′), 149.34 (C-3′), 145.52 (C-4), 140.92 (C-4′′′), 136.04 (C-1′′′′), 135.01 (C-5′′′′), 133.50 (C-1′′′), 132.87 (C-1′′), 130.50 (C-2′′′′), 130.07 (C-3′′′′), 129.72 (C-4′′), 129.48 (C-3′′ & C-5′′), 128.92 (C-3′′′ & C-5′′′), 127.65 (C-2′′′ & C-6′′′), 127.14 (C-2′′ & C-6′′), 127.06 (C-4′′′′), 125.28 (C-6′′′′), 102.23 (C-5), 35.67 (C-8′′′), 27.50 (C-6), 20.45 (CH_3_-5′′′′), 17.39 (CH_3_-2′′′′). Anal. calc. for C_28_H_26_N_6_OS_2_ (526.67): C, 63.85; H, 4.98; N, 15.96. Found: C, 63.82; H, 4.94; N, 15.95.

#### 3.3.3 4-[({5-[(2-Amino-1,3-thiazol-4-yl)methyl]-4-phenyl-4*H*-1,2,4-triazol-3-yl}sulfanyl)methyl]-*N*-(2,6-dimethylphenyl)benzamide (9c)

Mol. formula: C_28_H_26_N_6_OS_2_; mol. mass.: 526 g mol^−1^; IR (KBr, *ν*/cm^−1^): 3355 (N–H str.), 3060 (C–H str. of aromatic ring), 2928 (–CH_2_– str.), 1662 (C=O str.), 1571 (C=C str. of aromatic ring), 1527 (C=N str.), 1174 (C–N–C bond str.), 638 (C–S str.); ^1^H-NMR (600 MHz, DMSO-d_6_, *δ*/ppm): 9.75 (s, 1H, –CO–NH-1′′′′), 7.91 (br.d, *J* = 7.6, 2H, H-2′′′ & H-6′′′), 7.50–7.48 (m, 3H, H-3′′, H-4′′ & H-5′′), 7.45 (br.d, *J* = 7.9, 2H, H-3′′′ & H-5′′′), 7.23 (br.d, *J* = 6.7, 2H, H-2′′ & H-6′′), 7.13 (br.s, 3H, H-3′′′′, H-4′′′′ & H-5′′′′), 6.85 (br.s, 2H, H_2_N-2), 5.85 (s, 1H, H-5), 4.39 (s, 2H, CH_2_-8′′′), 3.77 (s, 2H, CH_2_-6), 2.18 (s, 6H, CH_3_-2′′′′ & CH_3_-6′′′′); ^13^C-NMR (150 MHz, DMSO-d_6_, *δ*/ppm): 168.21 (C-2), 164.61 (C-7′′′), 153.52 (C-5′), 149.32 (C-3′), 145.56 (C-4), 140.97 (C-4′′′), 135.59 (C-2′′′′ & C-6′′′′), 135.21 (C-1′′′′), 133.29 (C-1′′′), 132.90 (C-1′′), 129.70 (C-4′′), 129.45 (C-3′′ & C-5′′), 128.97 (C-3′′′ & C-5′′′), 127.67 (C-2′′′ & C-6′′′), 127.56 (C-3′′′′ & C-5′′′′), 127.15 (C-2′′ & C-6′′), 126.65 (C-4′′′′), 102.19 (C-5), 35.67 (C-8′′′), 27.52 (C-6), 17.99 (CH_3_-2′′′′ & CH_3_-6′′′′). Anal. calc. for C_28_H_26_N_6_OS_2_ (526.67): C, 63.85; H, 4.98; N, 15.96. Found: C, 63.89; H, 4.92; N, 15.98.

#### 3.3.4 4-[({5-[(2-Amino-1,3-thiazol-4-yl)methyl]-4-phenyl-4*H*-1,2,4-triazol-3-yl}sulfanyl)methyl]-*N*-(3,4-dimethylphenyl)benzamide (9d)

Mol. formula: C_28_H_26_N_6_OS_2_; mol. mass.: 526 g mol^−1^; IR (KBr, *ν*/cm^−1^): 3358 (N–H str.), 3040 (C–H str. of aromatic ring), 2912 (–CH_2_– str.), 1652 (C=O str.), 1551 (C=C str. of aromatic ring), 1511 (C=N str.), 1149 (C–N–C bond str.), 612 (C–S str.); ^1^H-NMR (600 MHz, DMSO-d_6_, *δ*/ppm): 10.05 (s, 1H, –CO–NH-1′′′′), 7.85 (br.d, *J* = 7.6, 2H, H-2′′′ & H-6′′′), 7.54 (br.s, 1H, H-2′′′′), 7.50–7.47 (m, 4H, H-3′′, H-4′′, H-5′′ & H-6′′′′), 7.44 (br.d, *J* = 7.8, 2H, H-3′′′ & H-5′′′), 7.24 (br.d, *J* = 6.7, 2H, H-2′′ & H-6′′), 7.09 (br.d, *J* = 8.0, 1H, H-5′′′′), 6.84 (br.s, 2H, H_2_N-2), 5.84 (s, 1H, H-5), 4.39 (s, 2H, CH_2_-8′′′), 3.77 (s, 2H, CH_2_-6), 2.22 (s, 3H, CH_3_-4′′′′), 2.19 (s, 3H, CH_3_-3′′′′); ^13^C-NMR (150 MHz, DMSO-d_6_, *δ*/ppm): 168.20 (C-2), 164.81 (C-7′′′), 153.49 (C-5′), 149.31 (C-3′), 145.56 (C-4), 140.82 (C-4′′′), 136.74 (C-3′′′′), 136.09 (C-1′′′′), 133.97 (C-1′′′), 132.91 (C-1′′), 131.39 (C-4′′′′), 129.71 (C-4′′), 129.48 (C-3′′ & C-5′′), 128.86 (C-3′′′ & C-5′′′), 127.64 (C-2′′′ & C-6′′′), 127.16 (C-2′′ & C-6′′), 121.60 (C-5′′′′), 117.90 (C-2′′′′), 102.20 (C-5), 35.66 (C-8′′′), 27.51 (C-6), 19.59 (CH_3_-3′′′′), 18.78 (CH_3_-4′′′′). Anal. calc. for C_28_H_26_N_6_OS_2_ (526.67): C, 63.85; H, 4.98; N, 15.96. Found: C, 63.80; H, 4.99; N, 15.99.

#### 3.3.5 4-[({5-[(2-Amino-1,3-thiazol-4-yl)methyl]-4-phenyl-4*H*-1,2,4-triazol-3-yl}sulfanyl)methyl]-*N*-(3,5-dimethylphenyl)benzamide (9e)

Mol. formula: C_28_H_26_N_6_OS_2_; mol. mass.: 526 g mol^−1^; IR (KBr, *ν*/cm^−1^): 3339 (N–H str.), 3040 (C–H str. of aromatic ring), 2934 (–CH_2_– str.), 1677 (C=O str.), 1562 (C=C str. of aromatic ring), 1518 (C=N str.), 1165 (C–N–C bond str.), 620 (C–S str.); ^1^H-NMR (600 MHz, DMSO-d_6_, *δ*/ppm): 10.00 (s, 1H, –CO–NH-1′′′′), 7.85 (br.d, *J* = 7.6, 2H, H-2′′′ & H-6′′′), 7.50–7.45 (m, 3H, H-3′′, H-4′′ & H-5′′), 7.45 (br.d, *J* = 7.7, 2H, H-3′′′ & H-5′′′), 7.40 (br.s, 2H, H-2′′′′ & H-6′′′′), 7.23 (br.d, *J* = 6.8, 2H, H-2′′ & H-6′′), 6.85 (br.s, 2H, H_2_N-2), 6.75 (br.s, 1H, H-4′′′′), 5.84 (s, 1H, H-5), 4.39 (s, 2H, CH_2_-8′′′), 3.77 (s, 2H, CH_2_-6), 2.20 (s, 6H, CH_3_-3′′′′ & CH_3_-5′′′′); ^13^C-NMR (150 MHz, DMSO-d_6_, *δ*/ppm): 168.21 (C-2), 164.97 (C-7′′′), 153.50 (C-5′), 149.32 (C-3′), 145.56 (C-4), 140.89 (C-4′′′), 138.88 (C-1′′′′), 137.48 (C-3′′′′), 133.96 (C-1′′′), 132.90 (C-1′′), 129.71 (C-4′′), 129.48 (C-3′′ & C-5′′), 128.88 (C-3′′′ & C-5′′′), 127.66 (C-2′′′ & C-6′′′), 127.15 (C-2′′ & C-6′′), 125.16 (C-4′′′′), 118.11 (C-2′′′′), 102.21 (C-5), 35.65 (C-8′′′), 27.51 (C-6), 21.08 (CH_3_–C-3′′′′& C-5′′′′). Anal. calc. for C_28_H_26_N_6_OS_2_ (526.67): C, 63.85; H, 4.98; N, 15.96. Found: C, 63.84; H, 4.96; N, 15.98.

#### 3.3.6 4-[({5-[(2-Amino-1,3-thiazol-4-yl)methyl]-4-phenyl-4*H*-1,2,4-triazol-3-yl}sulfanyl)methyl]-*N*-(2-methylphenyl)benzamide (9f)

Mol. formula: C_27_H_24_N_6_OS_2_; mol. mass.: 512 g mol^−1^; IR (KBr, *ν*/cm^−1^): 3361 (N–H str.), 3068 (C–H str. of aromatic ring), 2940 (–CH_2_– str.), 1688 (C=O str.), 1541 (C=C str. of aromatic ring), 1531 (C=N str.), 1178 (C–N–C bond str.), 634 (C–S str.); ^1^H-NMR (600 MHz, DMSO-d_6_, *δ*/ppm): 9.86 (s, 1H, –CO–NH-1′′′′), 7.90 (br.d, *J* = 8.1, 2H, H-2′′′ & H-6′′′), 7.50–7.47 (m, 3H, H-3′′, H-4′′ & H-5′′), 7.46 (br.d, *J* = 8.2, 2H, H-3′′′ & H-5′′′), 7.34 (br.d, *J* = 7.6, 1H, H-6′′′′), 7.27 (br.d, *J* = 7.3, 1H, H-3′′′′), 7.25–7.21 (m, 3H, H-2′′, H-6′′ & H-5′′′′), 7.16 (dis.t, *J* = 7.3 1H, H-4′′′′), 6.86 (br.s, 2H, H_2_N-2), 5.85 (s, 1H, H-5), 4.40 (s, 2H, CH_2_-8′′′), 3.78 (s, 2H, CH_2_-6), 2.23 (s, 3H, CH_3_-2′′′′); ^13^C-NMR (150 MHz, DMSO-d_6_, *δ*/ppm): 168.22 (C-2), 164.89 (C-7′′′), 153.52 (C-5′), 149.32 (C-3′), 145.57 (C-4), 140.98 (C-4′′′), 133.69 (C-1′′′), 133.52 (C-1′′′′), 133.46 (C-2′′′′), 132.90 (C-1′′), 130.26 (C-3′′′′), 129.71 (C-4′′), 129.47 (C-3′′ & C-5′′), 128.94 (3′′′ & C-5′′′), 127.70 (C-2′′′ & C-6′′′), 127.16 (C-2′′ & C-6′′), 126.57 (C-5′′′′), 125.96 (C-4′′′′), 125.29 (C-6′′′′), 102.20 (C-5), 35.66 (C-8′′′), 27.52 (C-6), 17.84 (CH_3_-2′′′′). Anal. calc. for C_27_H_24_N_6_OS_2_ (512.64): C, 63.26; H, 4.72; N, 16.39. Found: 63.31; H, 4.70; N, 16.36.

#### 3.3.7 4-[({5-[(2-Amino-1,3-thiazol-4-yl)methyl]-4-phenyl-4*H*-1,2,4-triazol-3-yl}sulfanyl)methyl]-*N*-(3-methylphenyl)benzamide (9g)

Mol. formula: C_27_H_24_N_6_OS_2_; mol. mass.: 512 g mol^−1^; IR (KBr, *ν*/cm^−1^): 3341 (N–H str.), 3053 (C–H str. of aromatic ring), 2921 (–CH_2_– str.), 1671 (C=O str.), 1562 (C=C str. of aromatic ring), 1516 (C=N str.), 1164 (C–N–C bond str.), 622 (C–S str.); ^1^H-NMR (600 MHz, DMSO-d_6_, *δ*/ppm): 10.14 (s, 1H, –CO–NH-1′′′′), 7.86 (br.d, *J* = 8.1, 2H, H-2′′′ & H-6′′′), 7.60 (br.s, 1H, H-2′′′′), 7.55 (br.d, *J* = 7.1, 1H, H-6′′′′), 7.52–7.49 (m, 3H, H-3′′, H-4′′ & H-5′′), 7.45 (br.d, *J* = 8.2, 2H, H-3′′′ & H-5′′′), 7.24–7.21 (m, 3H, H-2′′, H-6′′ & H-5′′′′), 6.92 (br.d, *J* = 7.2, 1H, H-4′′′′), 6.85 (br.s, 2H, H_2_N-2), 5.84 (s, 1H, H-5), 4.39 (s, 2H, CH_2_-8′′′), 3.77 (s, 2H, CH_2_-6), 2.30 (s, 3H, CH_3_-3′′′′); ^13^C-NMR (150 MHz, DMSO-d_6_, *δ*/ppm): 168.21 (C-2), 165.06 (C-7′′′), 153.50 (C-5′), 149.32 (C-3′), 145.54 (C-4), 140.94 (C-4′′′), 138.95 (C-1′′′′), 137.71 (C-3′′′′), 133.91 (C-1′′′), 132.89 (C-1′′), 129.72 (C-4′′), 129.49 (C-3′′ & C-5′′), 128.89 (C-3′′′ & C-5′′′), 128.39 (C-5′′′′), 127.68 (C-2′′′ & C-6′′′), 127.15 (C-2′′ & C-6′′), 124.35 (C-4′′′′), 120.87 (C-2′′′′), 117.52 (C-6′′′′), 102.22 (C-5), 35.64 (C-8′′′), 27.50 (C-6), 21.16 (CH_3_-3′′′′). Anal. calc. for C_27_H_24_N_6_OS_2_ (512.64): C, 63.26; H, 4.72; N, 16.39. Found: 63.29; H, 4.77; N, 16.34.

#### 3.3.8 4-[({5-[(2-Amino-1,3-thiazol-4-yl)methyl]-4-phenyl-4*H*-1,2,4-triazol-3-yl}sulfanyl)methyl]-*N*-(4-dimethylphenyl)benzamide (9h)

Mol. formula: C_27_H_24_N_6_OS_2_; mol. mass.: 512 g mol^−1^; IR (KBr, *ν*/cm^−1^): 3349 (N–H str.), 3054 (C–H str. of aromatic ring), 2922 (–CH_2_– str.), 1672 (C=O str.), 1561 (C=C str. of aromatic ring), 1517 (C=N str.), 1165 (C–N–C bond str.), 623 (C–S str.); ^1^H-NMR (600 MHz, DMSO-d_6_, *δ*/ppm): 10.13 (s, 1H, –CO–NH-1′′′′), 7.85 (br.d, *J* = 8.1, 2H, H-2′′′ & H-6′′′), 7.63 (br.d, *J* = 7.3, 2H, H-2′′′′ & H-6′′′′), 7.50–7.47 (m, 3H, H-3′′, H-4′′ & H-5′′), 7.44 (br.d, *J* = 8.2, 2H, H-3′′′ & H-5′′′), 7.22 (dd, *J* = 1.7, 8.1, 2H, H-2′′ & H-6′′), 7.14 (br.d, *J* = 8.2, 2H, H-3′′′′ & H-5′′′′), 6.84 (br.s, 2H, H_2_N-2), 5.83 (s, 1H, H-5), 4.39 (s, 2H, CH_2_-8′′′), 3.76 (s, 2H, CH_2_-6), 2.27 (s, 3H, CH_3_-4′′′′); ^13^C-NMR (150 MHz, DMSO-d_6_, *δ*/ppm): 168.20 (C-2), 164.94 (C-7′′′), 153.50 (C-5′), 149.31 (C-3′), 145.56 (C-4), 140.86 (C-4′′′), 136.53 (C-1′′′′), 133.95 (C-1′′′), 132.91 (C-1′′), 132.59 (C-4′′′′), 129.71 (C-4′′), 129.48 (C-3′′ & C-5′′), 128.90 (C-3′′′ & C-5′′′), 128.88 (C-3′′′), 127.66 (C-2′′′ & C-6′′′), 127.16 (C-2′′ & C-6′′), 120.20 (C-2′′′′), 102.20 (C-5), 35.65 (C-8′′′), 27.51 (C-6), 20.45 (CH_3_-4′′′′). Anal. calc. for C_27_H_24_N_6_OS_2_ (512.64): C, 63.26; H, 4.72; N, 16.39. Found: 63.23; H, 4.75; N, 16.36.

#### 3.3.9 4-[({5-[(2-Amino-1,3-thiazol-4-yl)methyl]-4-phenyl-4*H*-1,2,4-triazol-3-yl}sulfanyl)methyl]-*N*-(2-ethylphenyl)benzamide (9i)

Mol. formula: C_28_H_26_N_6_OS_2_; mol. mass.: 526 g mol^−1^; IR (KBr, *ν*/cm^−1^): 3354 (N–H str.), 3039 (C–H str. of aromatic ring), 2919 (–CH_2_– str.), 1680 (C=O str.), 1570 (C=C str. of aromatic ring), 1537 (C=N str.), 1162 (C–N–C bond str.), 625 (C–S str.); ^1^H-NMR (600 MHz, DMSO-d_6_, *δ*/ppm): 9.88 (s, 1H, –CO–NH-1′′′′), 7.90 (br.d, *J* = 8.2, 2H, H-2′′′ & H-6′′′), 7.52–7.49 (m, 4H, H-3′′, H-4′′, H-5′′ & H-6′′′′), 7.46 (br.d, *J* = 8.2, 2H, H-3′′′ & H-5′′′), 7.31–7.29 (m, 1H, H-3′′′′), 7.24–7.23 (m, 4H, H-2′′, H-6′′, H-4′′′′ & H-5′′′′), 6.86 (br.s, 2H, H_2_N-2), 5.85 (s, 1H, H-5), 4.40 (s, 2H, CH_2_-8′′′), 3.78 (s, 2H, CH_2_-6), 2.61 (q, 2H, *J* = 7.5, 
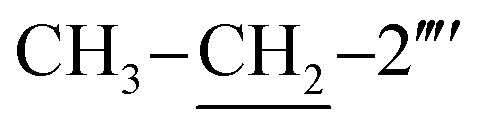
), 1.12 (t, 3H, *J* = 7.5, 
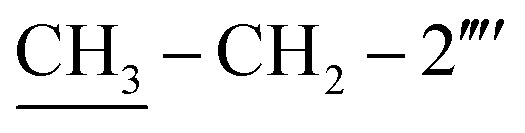
); ^13^C-NMR (150 MHz, DMSO-d_6_, *δ*/ppm): 168.22 (C-2), 165.27 (C-7′′′), 153.52 (C-5′), 149.33 (C-3′), 145.56 (C-4), 140.98 (C-4′′′), 139.82 (C-1′′′′), 135.67 (C-2′′′′), 133.44 (C-1′′′), 132.90 (C-1′′), 129.71 (C-4′′), 129.47 (C-3′′ & C-5′′), 128.99 (C-3′′′ & C-5′′′), 128.47 (C-3′′′′), 127.65 (C-2′′′ & C-6′′′), 127.55 (C-5′′′′), 127.16 (C-2′′ & C-6′′), 126.49 (C-4′′′′), 125.98 (C-6′′′′), 102.21 (C-5), 35.66 (C-8′′′), 27.52 (C-6), 23.96 (
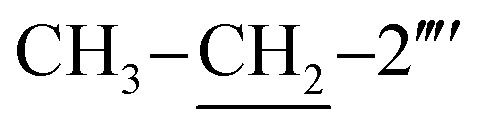
), 14.11 (
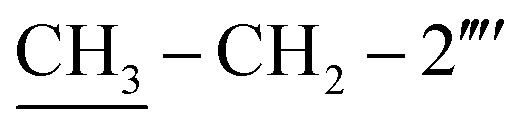
). Anal. calc. for C_28_H_26_N_6_OS_2_ (526.67): C, 63.85; H, 4.98; N, 15.96. Found: C, 63.80; H, 4.95; N, 15.93.

#### 3.3.10 4-[({5-[(2-Amino-1,3-thiazol-4-yl)methyl]-4-phenyl-4*H*-1,2,4-triazol-3-yl}sulfanyl)methyl]-*N*-(4-ethylphenyl)benzamide (9j)

Mol. formula: C_28_H_26_N_6_OS_2_; mol. mass.: 542 g mol^−1^; IR (KBr, *ν*/cm^−1^): 3344 (N–H str.), 3050 (C–H str. of aromatic ring), 2927 (–CH_2_– str.), 1677 (C=O str.), 1567 (C=C str. of aromatic ring), 1526 (C=N str.), 1180 (C–N–C bond str.), 631 (C–S str.); ^1^H-NMR (600 MHz, DMSO-d_6_, *δ*/ppm): 9.87 (s, 1H, –CO–NH-1′′′′), 7.89 (d, *J* = 7.9, 2H, H-2′′′ & H-6′′′), 7.51–7.49 (m, 3H, H-3′′, H-4′′ & H-5′′), 7.31–7.28 (m, 2H, H-2′′′′, H-6′′′′), 7.24–7.23 (m, 4H, H-2′′, H-6′′, H-3′′′′& H-5′′′′), 6.86 (br.s, 2H, H_2_N-2), 5.84 (s, 1H, H-5), 4.39 (s, 2H, CH_2_-8′′′), 3.77 (s, 2H, CH_2_-6), 2.61 (q, 2H, *J* = 7.4, 
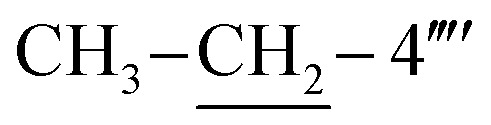
), 1.12 (t, 3H, *J* = 7.5, 
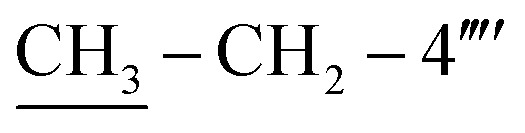
); ^13^C–N′MR (150 MHz, DMSO-d_6_, *δ*/ppm): 168.21 (C-2), 165.26 (C-7′′′), 153.51 (C-5′), 149.31 (C-3′), 145.56 (C-4), 140.97 (C-4′′′), 139.82 (C-4′′′′), 135.67 (C-1′′′′), 133.44 (C-1′′′), 132.91 (C-1′′), 129.70 (C-4′′), 129.47 (C-3′′ & C-5′′), 128.95 (C-3′′′ & C-5′′′), 128.47 (C-3′′′′), 127.65 (C-2′′′ & C-6′′′), 127.16 (C-2′′ & C-6′′), 125.98 (C-2′′′′), 102.20 (C-5), 35.66 (C-8′′′), 27.52 (C-6), 23.96 (
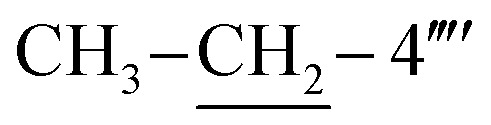
), 14.10 (
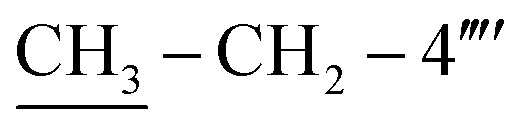
). Anal. calc. for C_28_H_26_N_6_O_2_S_2_ (542.67): C, 63.85; H, 4.98; N, 15.96. Found: C, 63.90; H, 4.96; N, 15.91.

#### 3.3.11 4-[({5-[(2-Amino-1,3-thiazol-4-yl)methyl]-4-phenyl-4*H*-1,2,4-triazol-3-yl}sulfanyl)methyl]-*N*-(3-ethoxyphenyl)benzamide (9k)

Mol. formula: C_28_H_26_N_6_O_2_S_2_; mol. mass.: 542 g mol^−1^; IR (KBr, *ν*/cm^−1^): 3361 (N–H str.), 3069 (C–H str. of aromatic ring), 2942 (–CH_2_– str.), 1684 (C=O str.), 1547 (C=C str. of aromatic ring), 1505 (C=N str.), 1141 (C–N–C bond str.), 608 (C–S str.); ^1^H-NMR (600 MHz, DMSO-d_6_, *δ*/ppm): 10.18 (s, 1H, –CO–NH-1′′′′), 7.95 (br.s, 1H, H-2′′′′), 7.87 (d, *J* = 8.1, 2H, H-2′′′ & H-6′′′), 7.51–7.49 (m, 3H, H-3′′, H-4′′ & H-5′′), 7.46 (br.d, *J* = 8.4, 2H, H-3′′′ & H-5′′′), 7.34 (br.d, *J* = 7.9, 1H, H-6′′′′), 7.24–7.22 (m, 3H, H-2′′, H-6′′ & H-5′′′′), 6.66 (dd, *J* = 1.7, 8.1, 1H, H-4′′′′), 6.85 (br.s, 2H, H_2_N-2), 5.84 (s, 1H, H-5), 4.40 (s, 2H, CH_2_-8′′′), 3.77 (s, 2H, CH_2_-6), 4.01 (q, 2H, *J* = 6.9, 
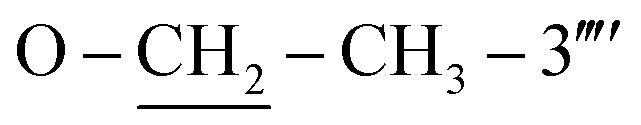
), 1.34 (t, 3H, *J* = 6.9, 
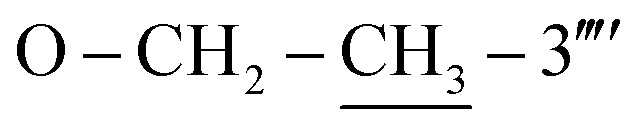
); ^13^C-NMR (150 MHz, DMSO-d_6_, *δ*/ppm): 168.22 (C-2), 165.16 (C-7′′′), 158.63 (C-3′′′′), 153.51 (C-5′), 149.32 (C-3′), 145.55 (C-4), 141.01 (C-4′′′), 140.24 (C-1′′′′), 133.89 (C-1′′′), 132.90 (C-1′′), 129.72 (C-4′′), 129.49 (C-3′′ & C-5′′), 129.32 (C-5′′′′), 128.90 (C-3′′′ & C-5′′′), 127.71 (C-2′′′ & C-6′′′), 127.15 (C-2′′ & C-6′′), 112.41 (C-6′′′′), 109.70 (C-4′′′′), 106.47 (C-2′′′′), 102.23 (C-5), 62.91(
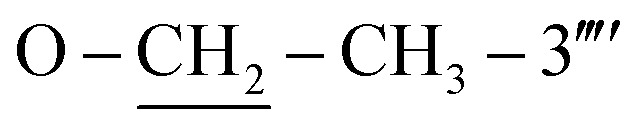
), 35.64 (C-8′′′), 27.51 (C-6), 14.63 (
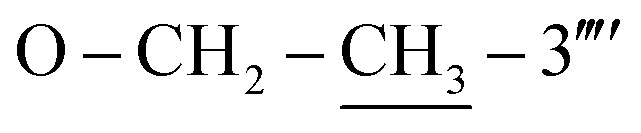
). Anal. calc. for C_28_H_26_N_6_O_2_S_2_ (542.67): C, 61.97; H, 4.83; N, 15.49. Found: C, 61.93; H, 4.80; N, 15.44.

### 3.4 Mushroom tyrosinase inhibition assay

The mushroom tyrosinase (Sigma Chemical, USA) inhibition was performed following our previously reported methods.^38–40^ In detail, 140 μL of phosphate buffer (20 mM, pH 6.8), 20 μL of mushroom tyrosinase (30 U mL^−1^) and 20 μL of the inhibitor solution were placed in the wells of a 96-well microplate. After pre-incubation for 10 minutes at room temperature, 20 μL of l-DOPA (3,4-dihydroxyphenylalanine, Sigma Chemical, USA) (0.85 mM) was added and the assay plate was further incubated at 25 °C for 20 minutes. Afterward, the absorbance of dopachrome was measured at 475 nm using a microplate reader (OPTI Max, Tunable). Kojic acid was used as a reference inhibitor and phosphate buffer was used as a negative control. The amount of inhibition by the test compounds was expressed as the percentage of concentration necessary to achieve 50% inhibition (IC_50_). Each concentration was analyzed in three independent experiments. IC_50_ values were calculated by nonlinear regression using GraphPad Prism 5.0.

The % inhibition of tyrosinase was calculated as following:Inhibition (%) = [(*B* − *S*)/*B*] × 100here, the *B* and *S* are the absorbance's for the blank and samples.

### 3.5 Kinetic analysis of the inhibition of tyrosinase

Based upon IC_50_ results, the most potent 9c compound was designated for the kinetic analysis. A series of experiments were performed to determine the inhibition kinetics of 9c by following the already reported methods.^41,42^ The inhibitor concentrations for 9c were 0.00, 0.004, 0.008, 0.016 and 0.032 μM. Substrate l-DOPA concentrations were between 0.125 and 2 mM in all kinetic studies. Pre-incubation and measurement time was the same as discussed in the mushroom tyrosinase inhibition assay protocol. Maximal initial velocity was determined from the initial linear portion of absorbance up to five minutes after addition of enzyme at a 30 s interval. The inhibition type of the enzyme was assayed by Lineweaver–Burk plots of the inverse of velocities (1/*V*) *versus* the inverse of substrate concentration 1/[l-DOPA] mM^−1^. The EI dissociation constant *K*_i_ was determined by the secondary plot of 1/*V versus* inhibitors concentrations.

### 3.6 Hemolytic activity

Bovine blood sample was collected in EDTA that was diluted with saline (0.9% NaCl), and centrifuge at 1000 × *g* for 10 min. The erythrocytes separated diluted in phosphate buffer saline of pH 7.4 and a suspension was made. Add 20 μL of synthetic compounds solution (10 mg mL^−1^) in 180 μL of RBCs suspension and incubate for 30 min at room temperature. PBS was used as negative control and Triton 100-X was taken as positive control.^43,44^ The percentage of hemolysis was taken as by using formula:
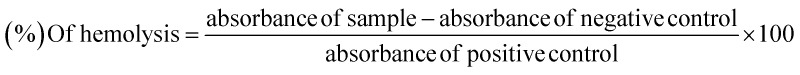


### 3.7 Molecular docking process

The crystal structure of mushroom tyrosinase was accessed from the protein data bank (PDB) having PDBID 2Y9X (https://www.rcsb.org/). Energy minimization of mushroom tyrosinase was carried out by using conjugate gradient algorithm and Amber force field in UCSF Chimera 1.10.1.^45^ The stereo-chemical properties, Ramachandran graph and values^46^ of mushroom tyrosinase structure were assessed by Molprobity server,^47^ while the hydrophobicity graph was generated by Discovery Studio 4.1 Client (https://discover.3ds.com/discovery-studio-visualizer-download). The protein architecture and statistical percentage values of helices, beta-sheets, coils and turns were accessed by using online tool VADAR 1.8 (https://vadar.wishartlab.com/).

## 4. Conclusions

A structurally distinctive series of novel molecules, amalgamated with a thiazole, a phenyl triazole and a benzamide moiety, was synthesized and recognized with very superb mushroom tyrosinase inhibition as compared to the standard used and most of previously reported inhibitors. It was postulated from their SAR studies that molecules, particularly bearing medium-sized non-polar group with +I effect at *ortho*-position in aryl part of the compound, generally inhibited the mushroom tyrosinase in an excellent manner. All these molecules also showed mild cytotoxicity towards red blood cell membranes. Therefore, it was concluded generally that these hybrid molecules might be pondered as creditable medicinal scaffolds for the treatment of tyrosinase related ailments, particularly skin disorders.

## Conflicts of interest

The authors declare no conflict of interest.

## Supplementary Material

RA-014-D4RA01063A-s001
