# Supplementary material for: Design of potent tyrosinase inhibiting N-arylated-4-yl-benzamides bearing 2-aminothiazole-triazole bi-heterocycles: mechanistic insight through enzyme inhibition, kinetics and computational studies
Source: RSC Adv. 2024 May 21;14(23):16546–59. doi: 10.1039/d4ra01063a (PMC11106707; doi:10.1039/d4ra01063a)
Supplement: RA-014-D4RA01063A-s001 [file RA-014-D4RA01063A-s001.pdf]

Electronic Supplementary Material (ESI) for RSC Advances.
This journal is © The Royal Society of Chemistry 2024

Designing of potent Tyrosinase Inhibiting *N*-Arylated-4-yl-benzamides bearing 2-aminothiazole-triazole bi-heterocycles: Mechanistic Insight through Enzyme Inhibition, Kinetics and Computational Studies.

Farhan Mahmood Khan[1], Muhammad Athar Abbasi[1,*], Aziz-ur-Rehman[1], Sabahat Zahra Siddiqui[1], Abdul Rehman Sadiq Butt[1], Hussain Raza[2], Mubashir Hassan[2], Syed Adnan Ali Shah[3], Muhammad Shahid[4] and Song Ja Kim[2]

[1]Department of Chemistry, Government College University, Lahore-54000, Pakistan.

[2]College of Natural Sciences, Department of Biological Science, Kongju National University, Gongju, 32588, South Korea.

[3]Faculty of Pharmacy and Atta-ur-Rahman Institute for Natural Products Discovery (AuRIns), Level 9, FF3, UniversitiTeknologi MARA, PuncakAlam Campus, 42300 Bandar PuncakAlam, Selangor DarulEhsan, Malaysia.

[4]Department of Biochemistry, University of Agriculture, Faisalabad-38040, Pakistan.

*Corresponding Authors: Dr. Muhammad Athar Abbasi, E-mail: abbasi@gcu.edu.pk Tel: (+92)-42-111000010 Ext. 266

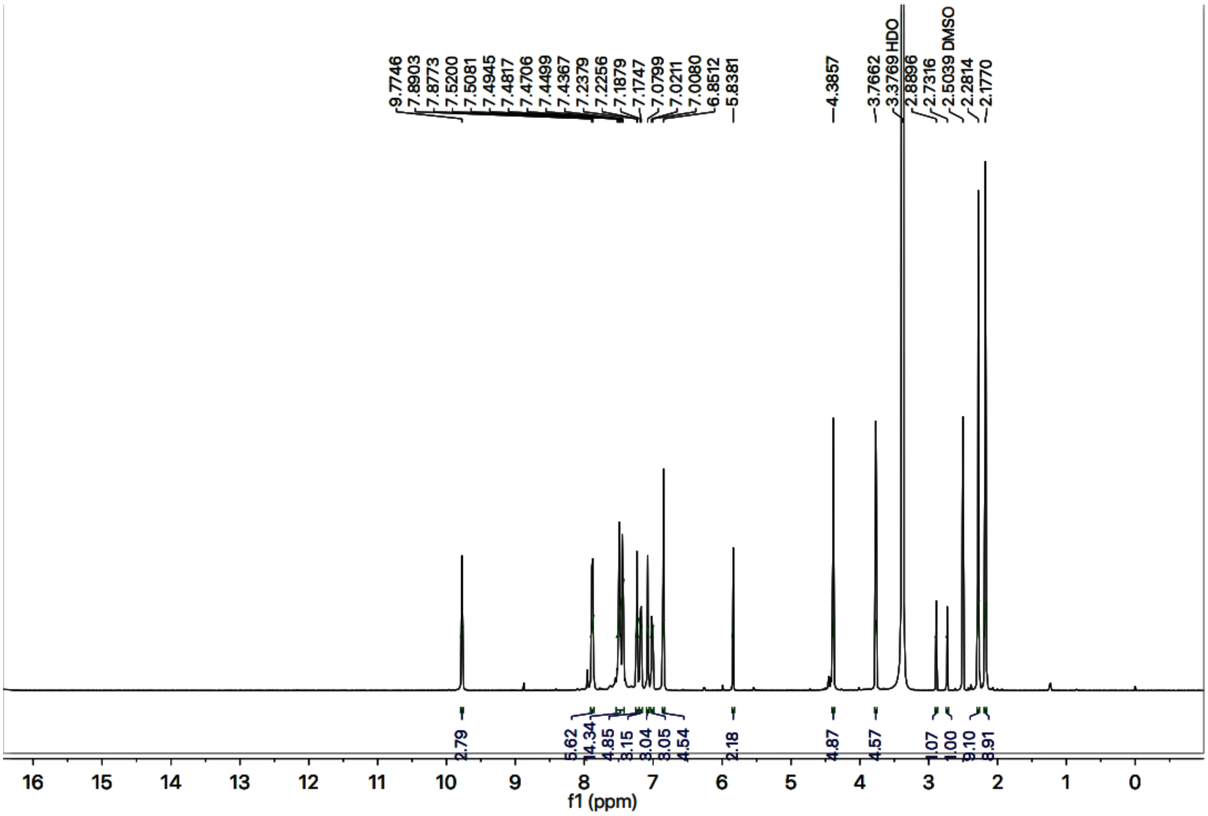

FIGURE S1 ^{1}H-NMR spectrum of **9a**

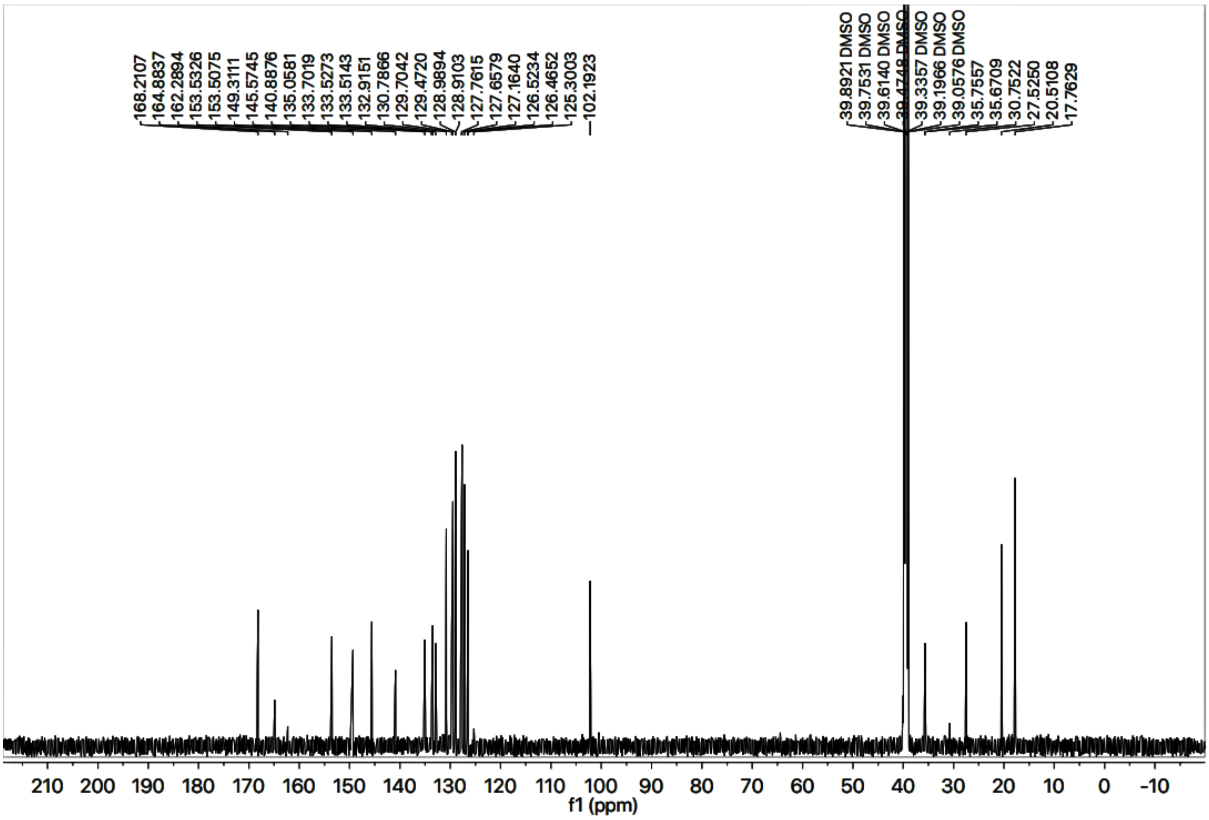

FIGURE S2 ^{13}C-NMR spectrum of **9a**

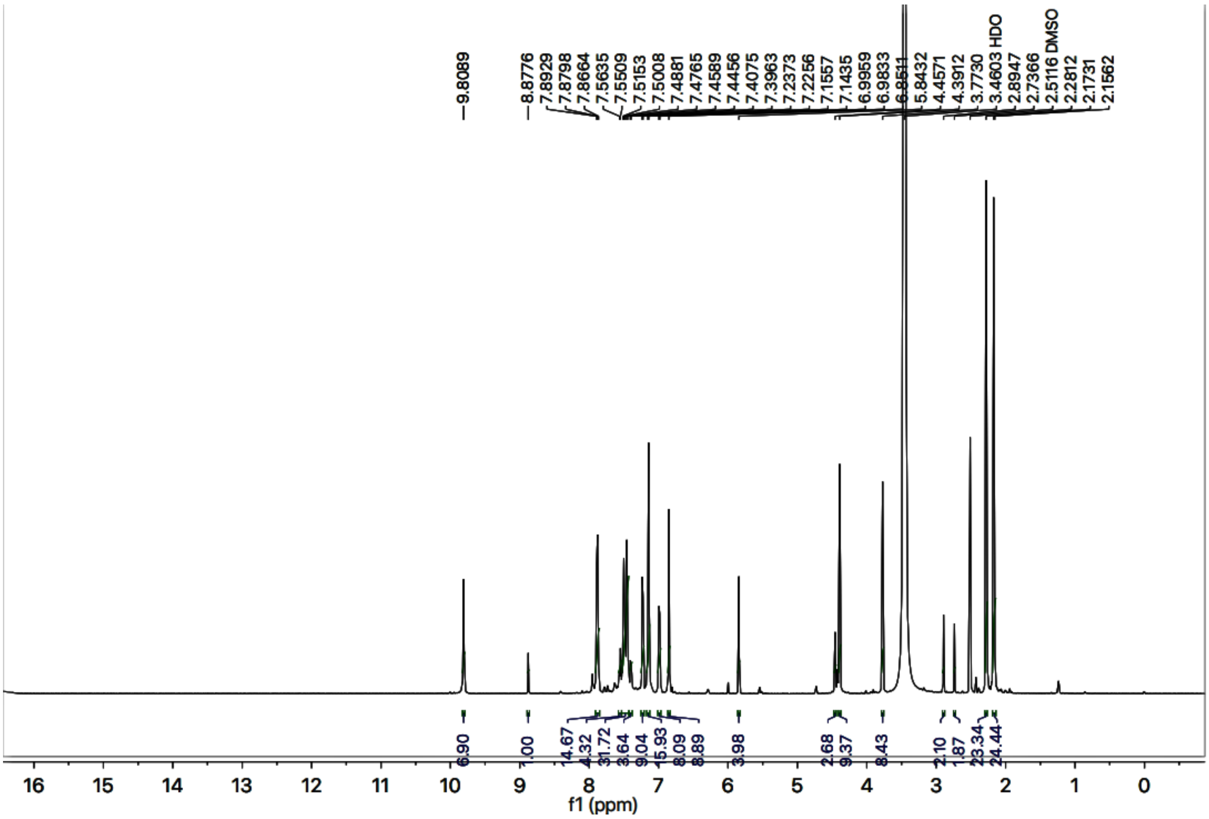

FIGURE S3 ^{1}H-NMR spectrum of **9b**

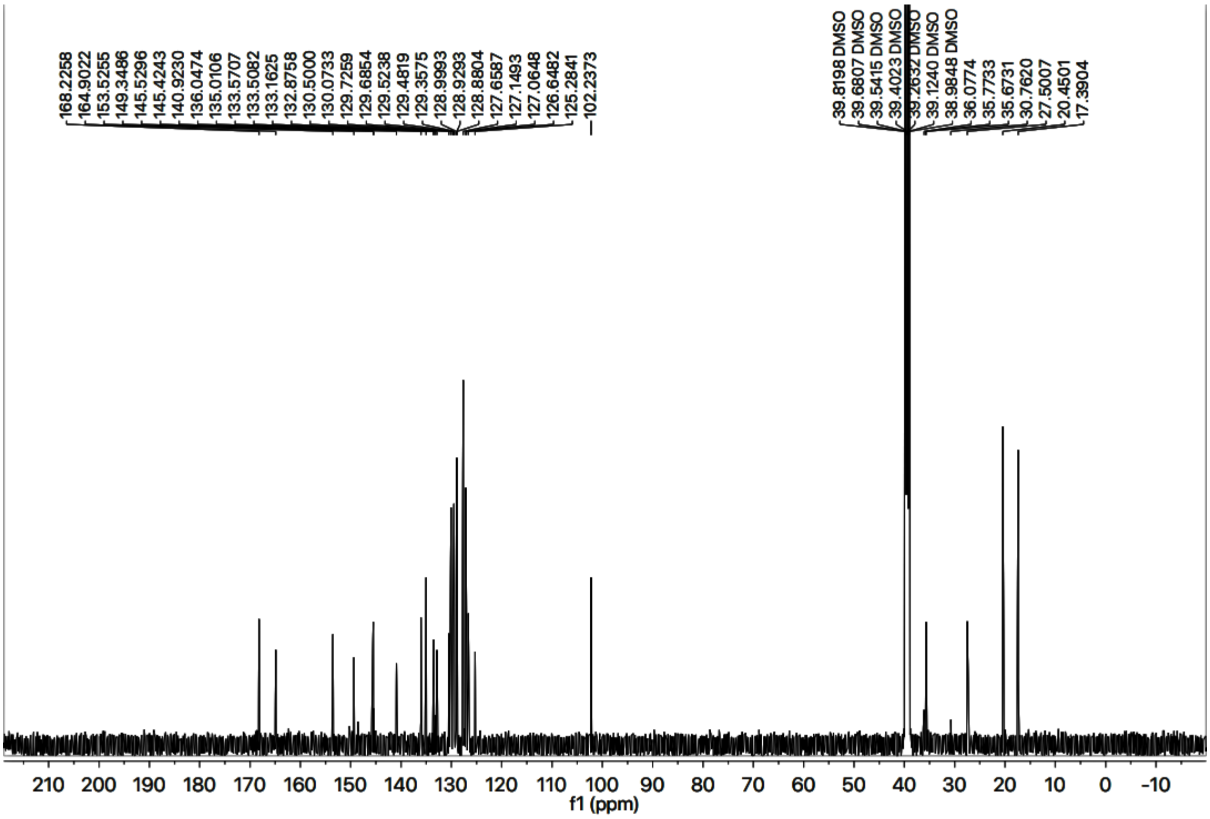

FIGURE S4 ^{13}C-NMR spectrum of **9b**

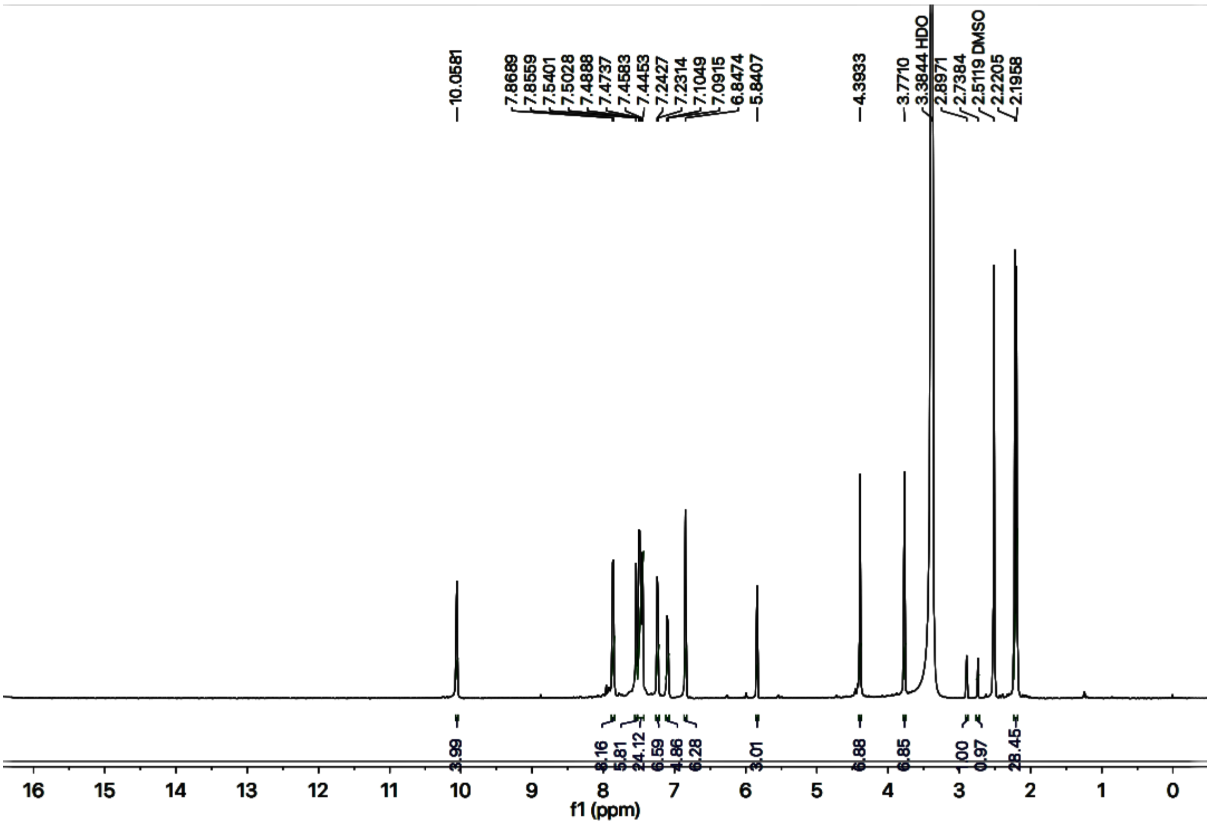

FIGURE S5 ^{1}H-NMR spectrum of **9d**

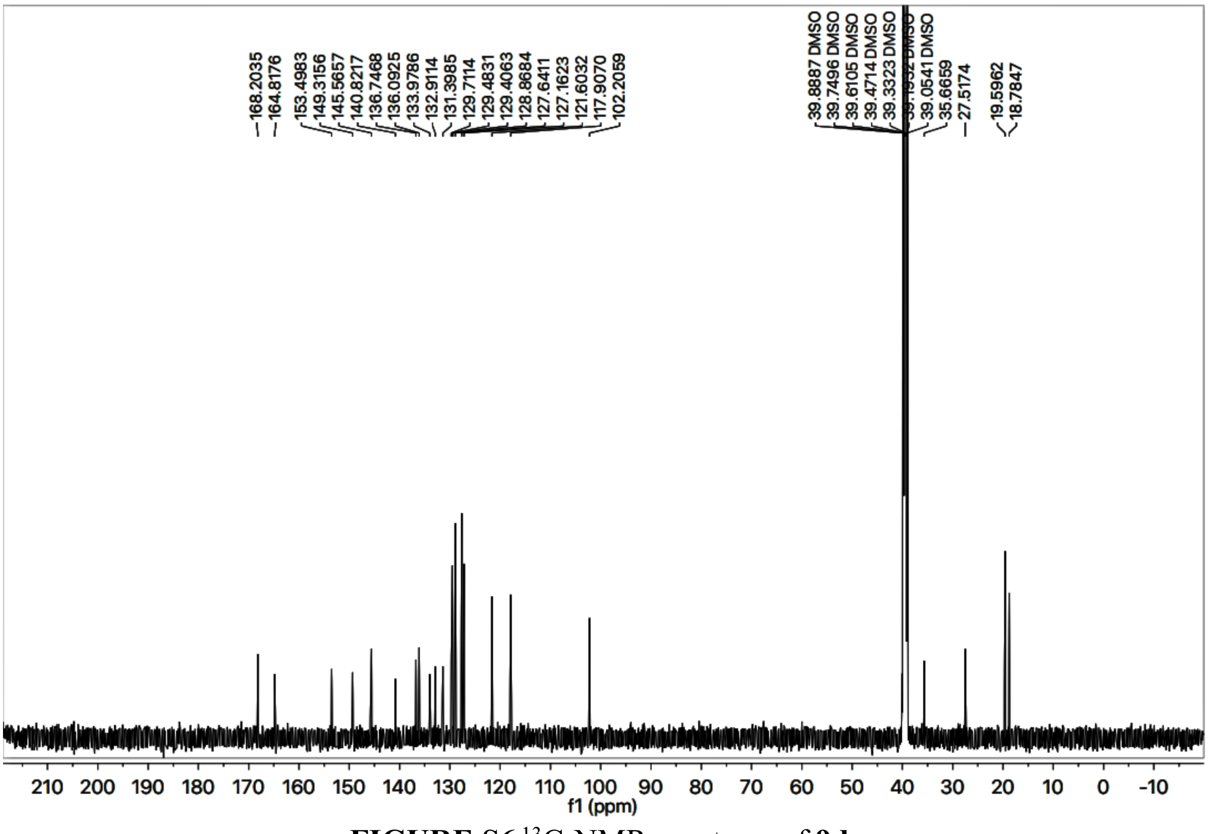

FIGURE S6 ^{13}C-NMR spectrum of **9d**

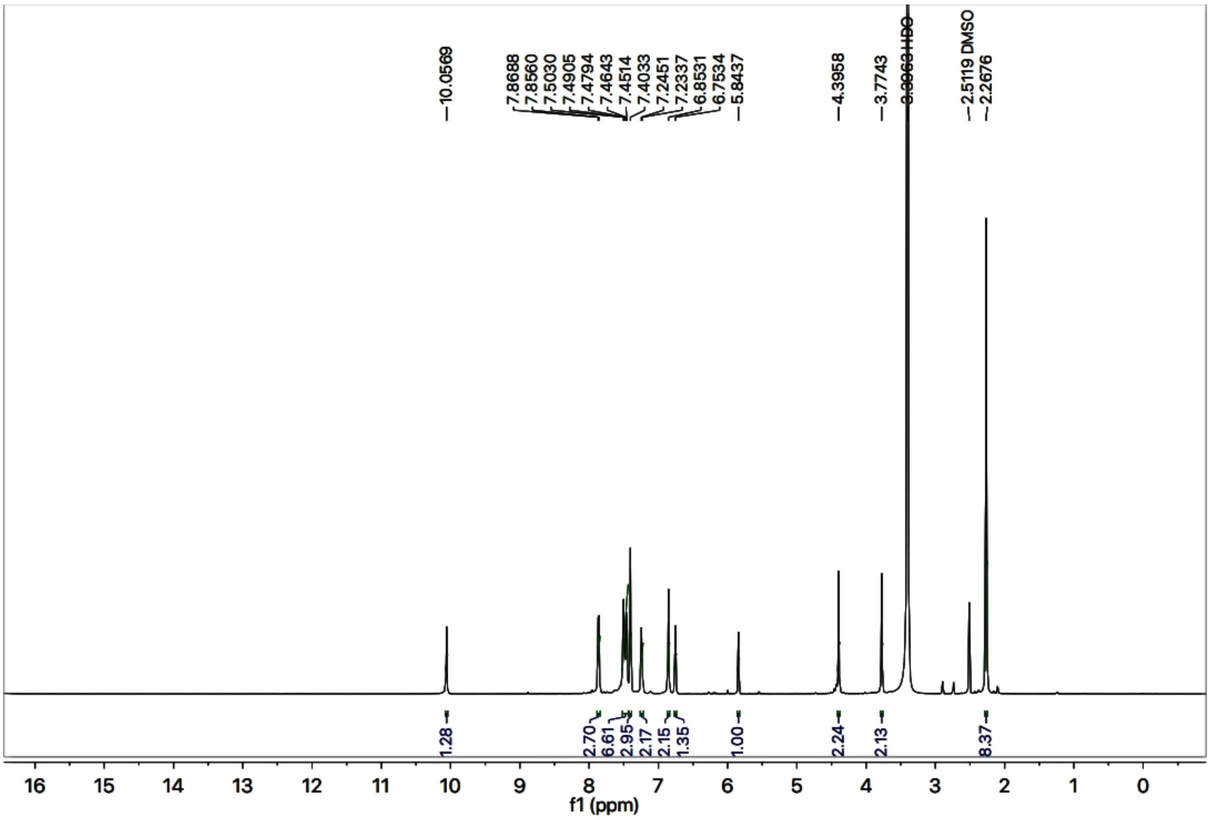

FIGURE S7 ^{1}H-NMR spectrum of **9e**

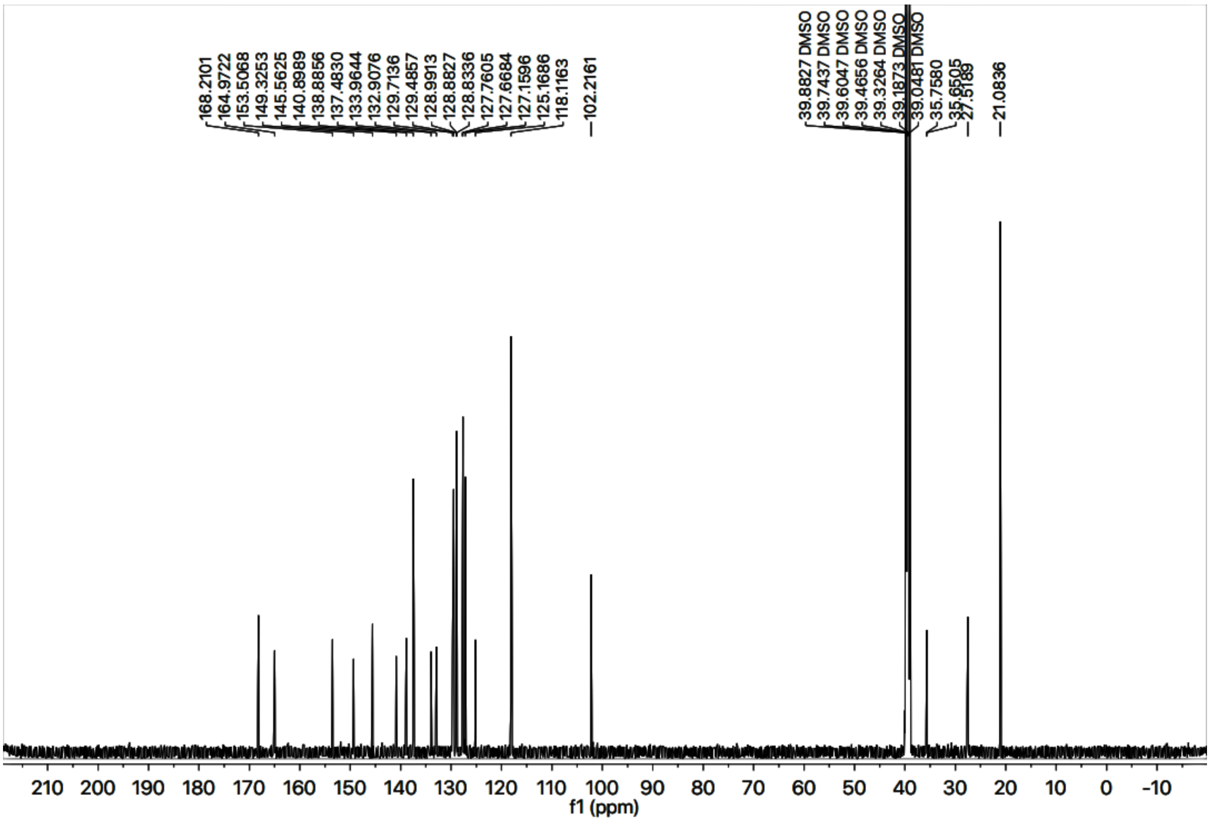

FIGURE S8 ^{13}C-NMR spectrum of **9e**

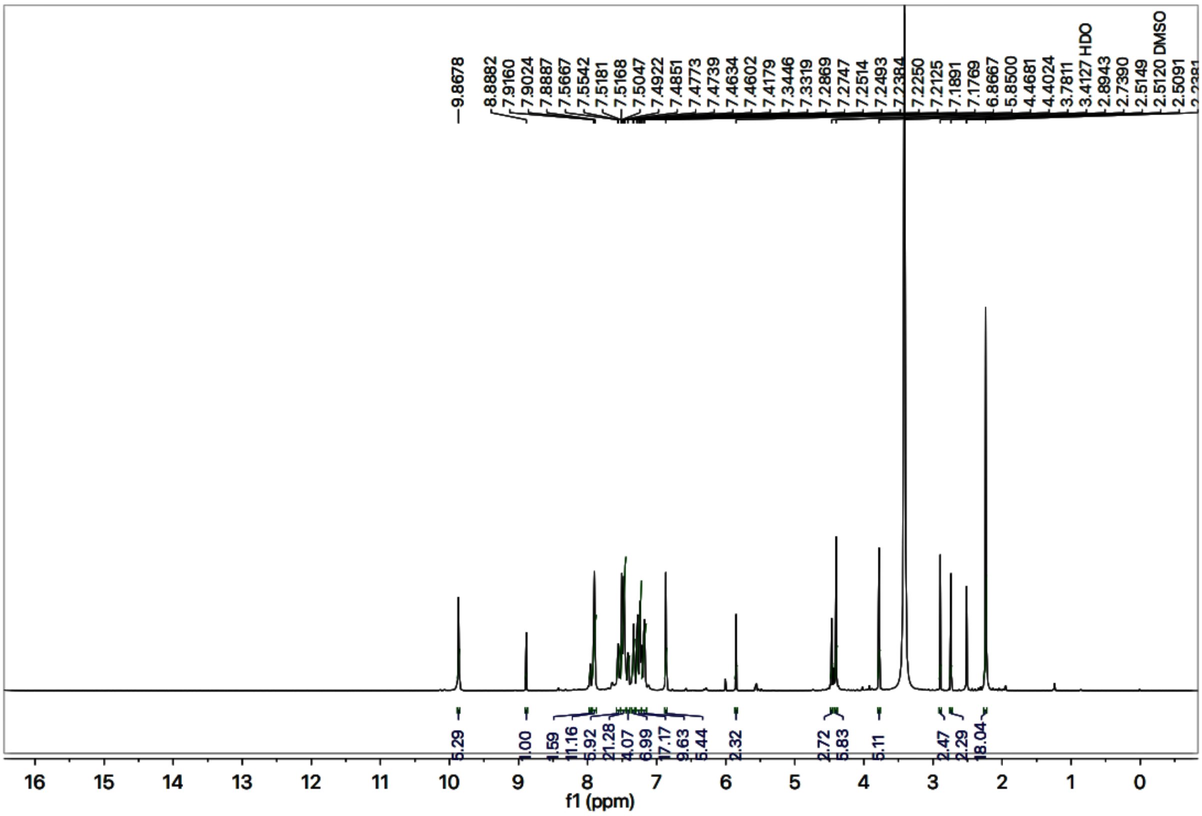

FIGURE S9 ^{1}H-NMR spectrum of **9f**

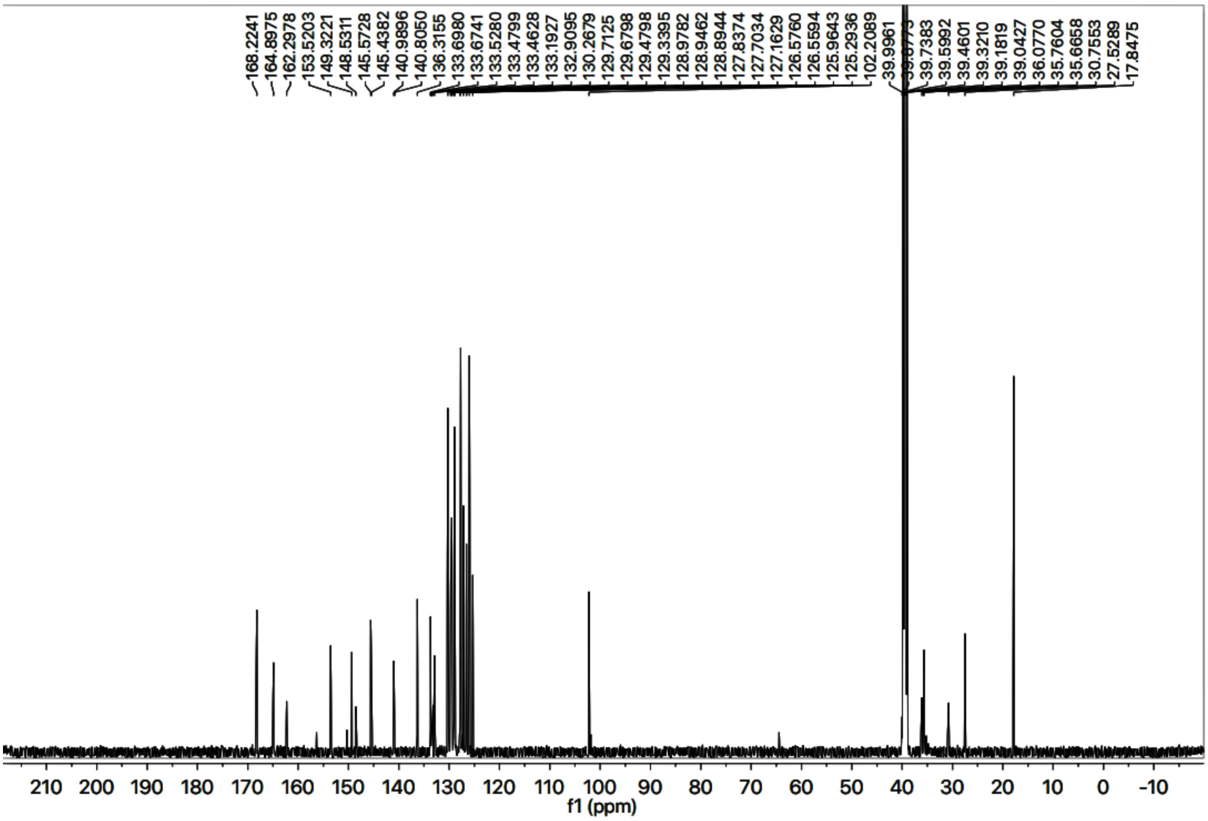

FIGURE S10 ^{13}C-NMR spectrum of **9f**

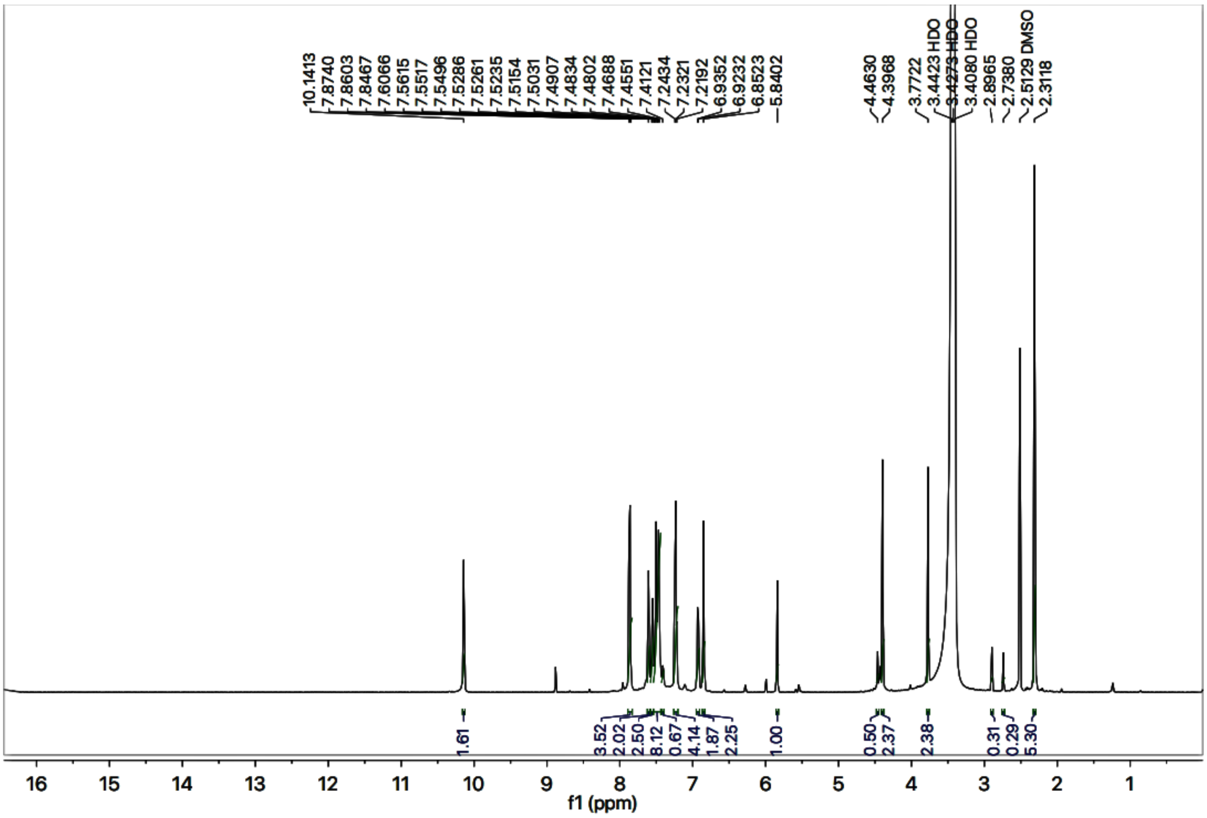

FIGURE S11 ^{1}H-NMR spectrum of **9g**

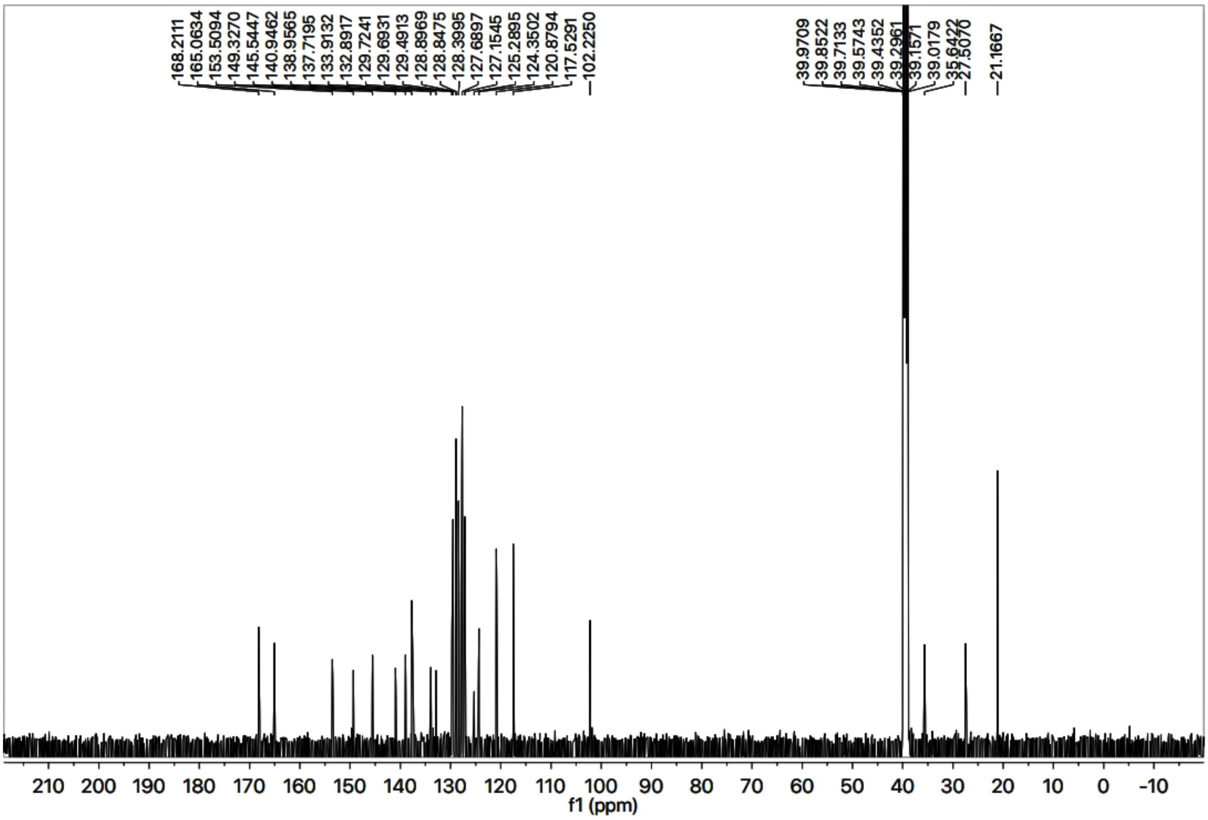

FIGURE S12 ^{13}C-NMR spectrum of **9g**

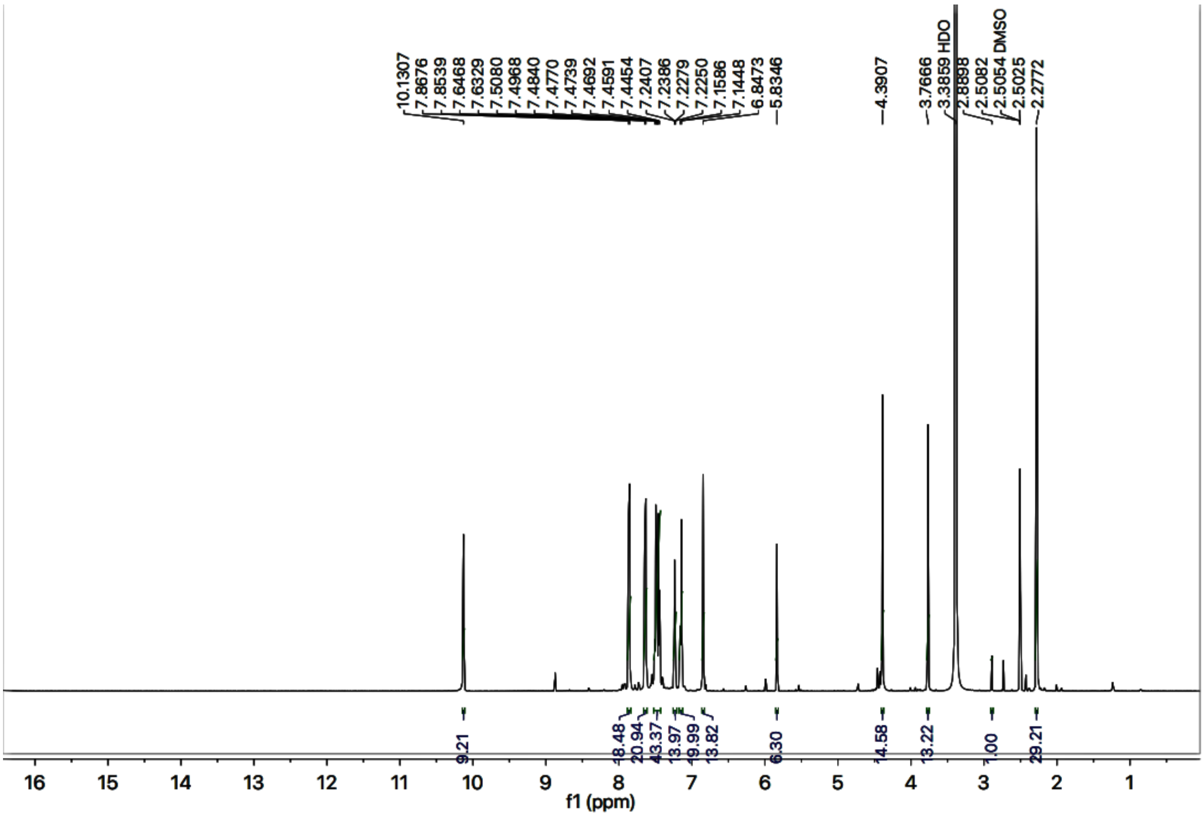

FIGURE S13 ^{1}H-NMR spectrum of **9h**

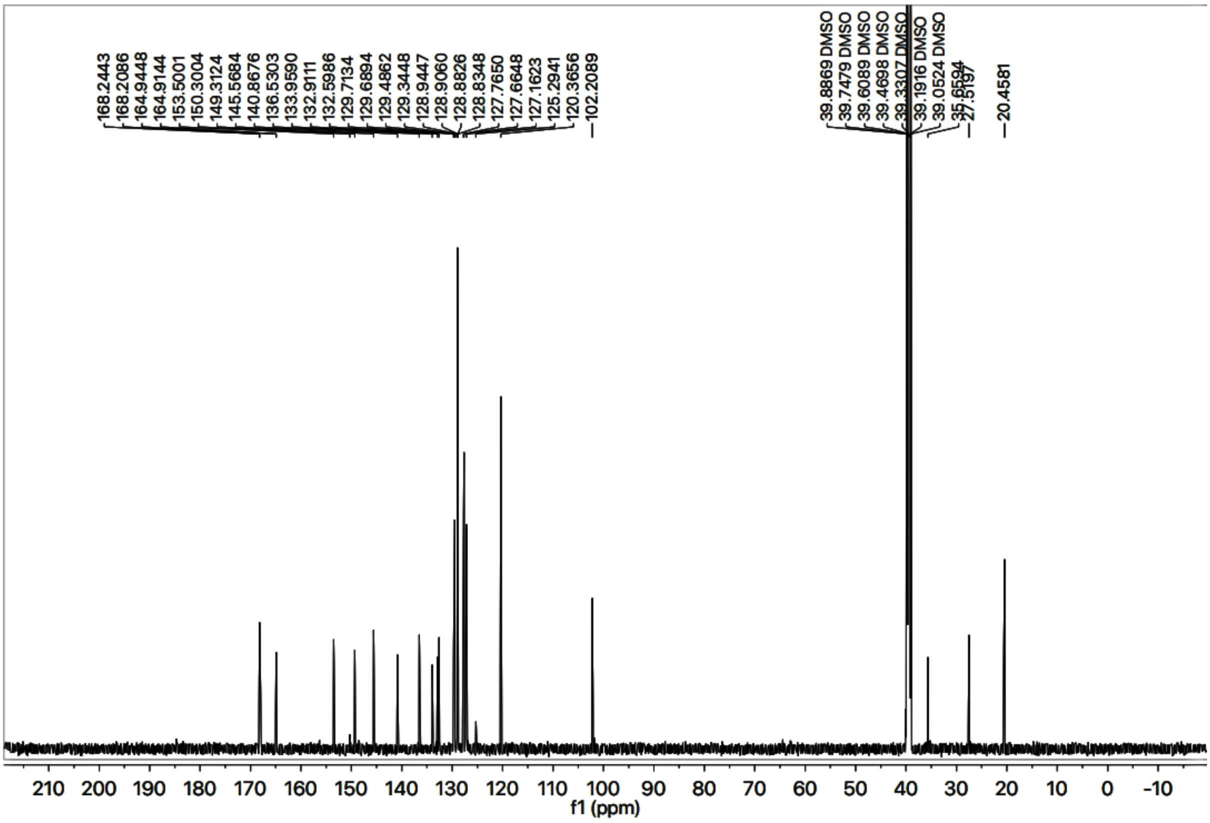

FIGURE S14 ^{13}C-NMR spectrum of **9h**

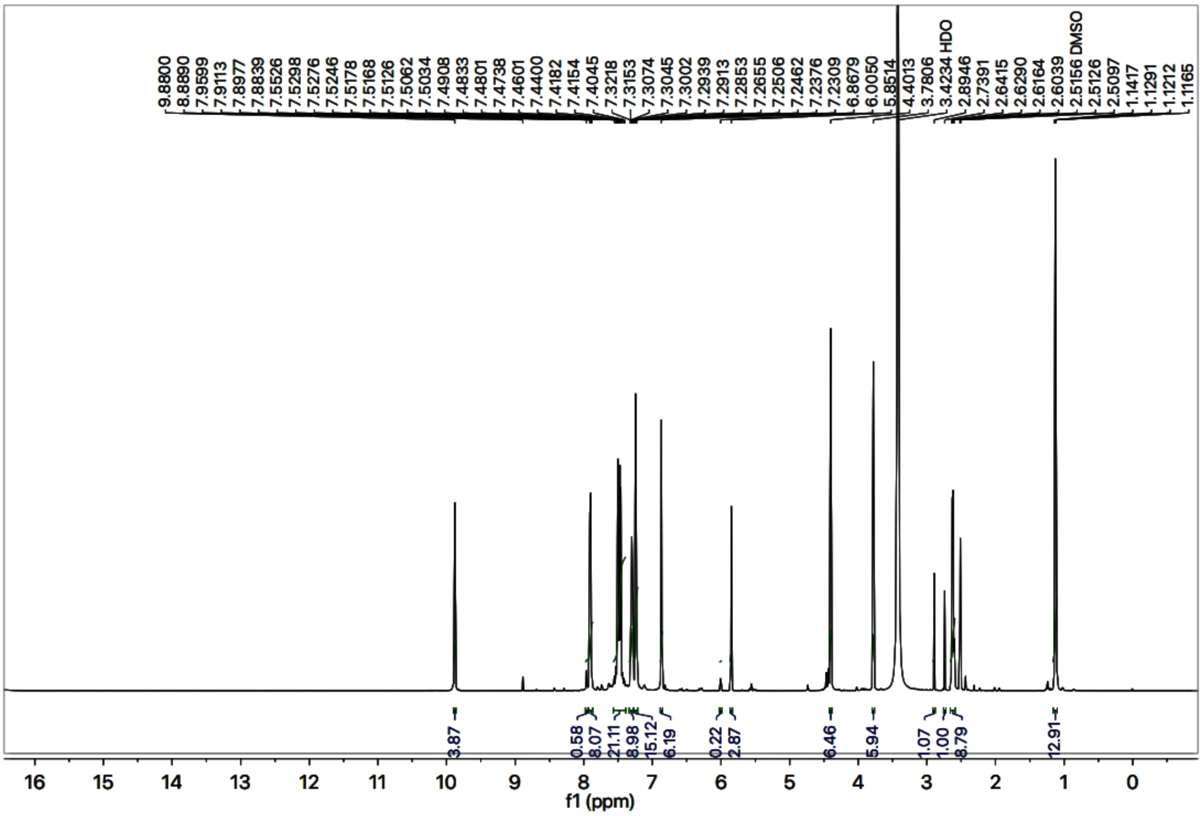

FIGURE S15 ^{1}H-NMR spectrum of **9i**

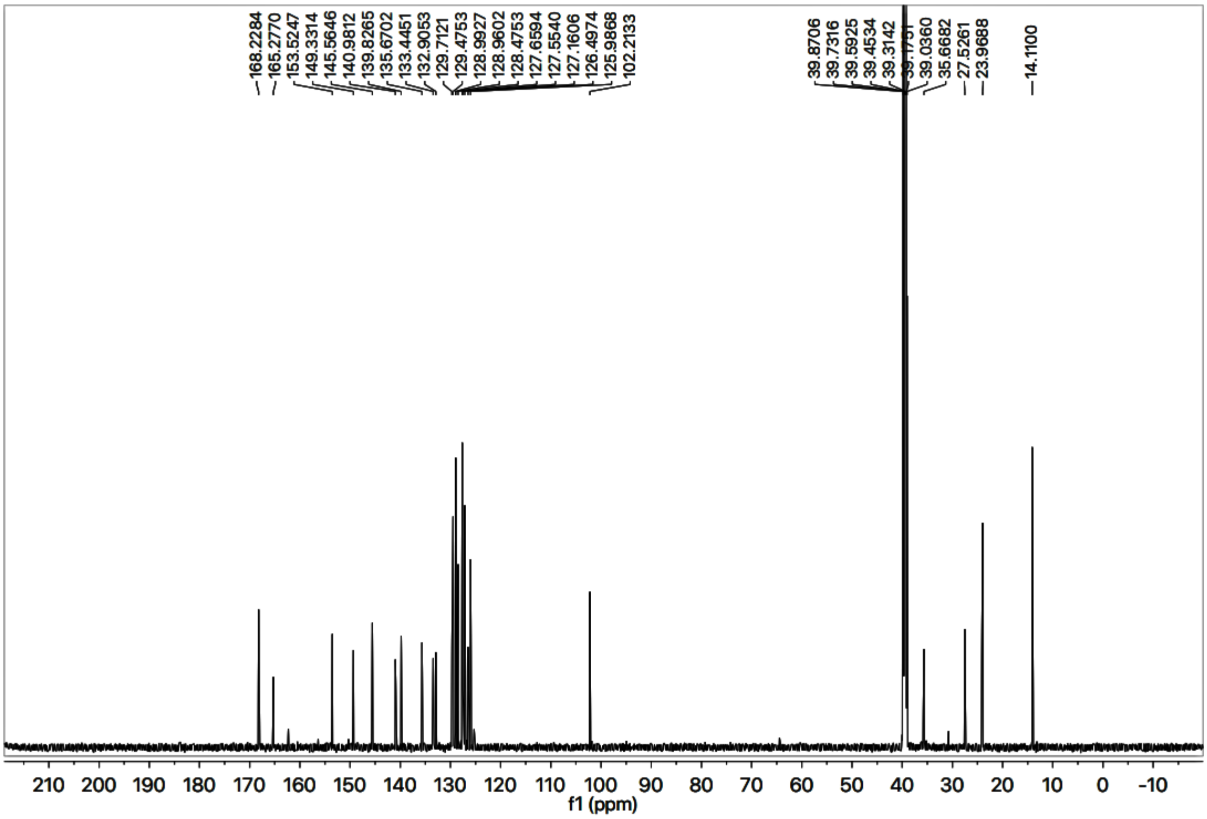

FIGURE S16 ^{13}C-NMR spectrum of **9i**

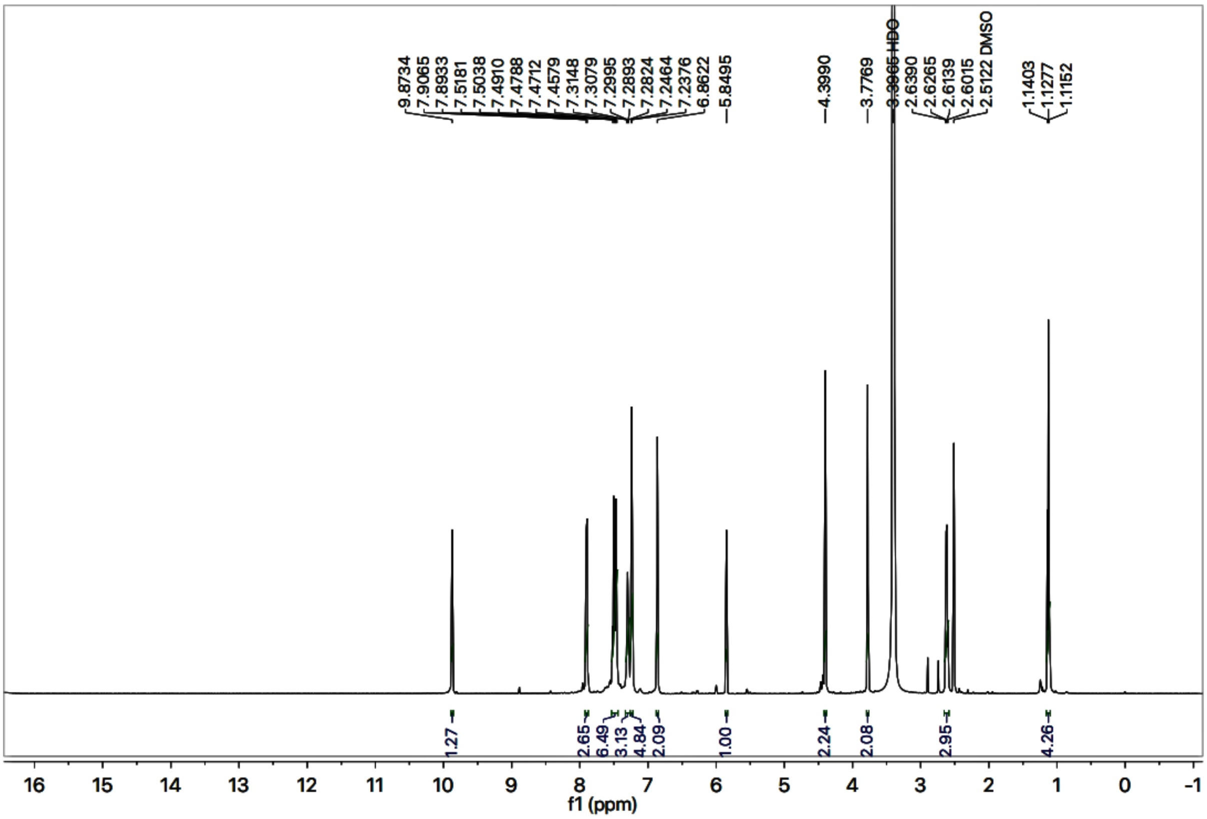

FIGURE S17 ^{1}H-NMR spectrum of **9j**

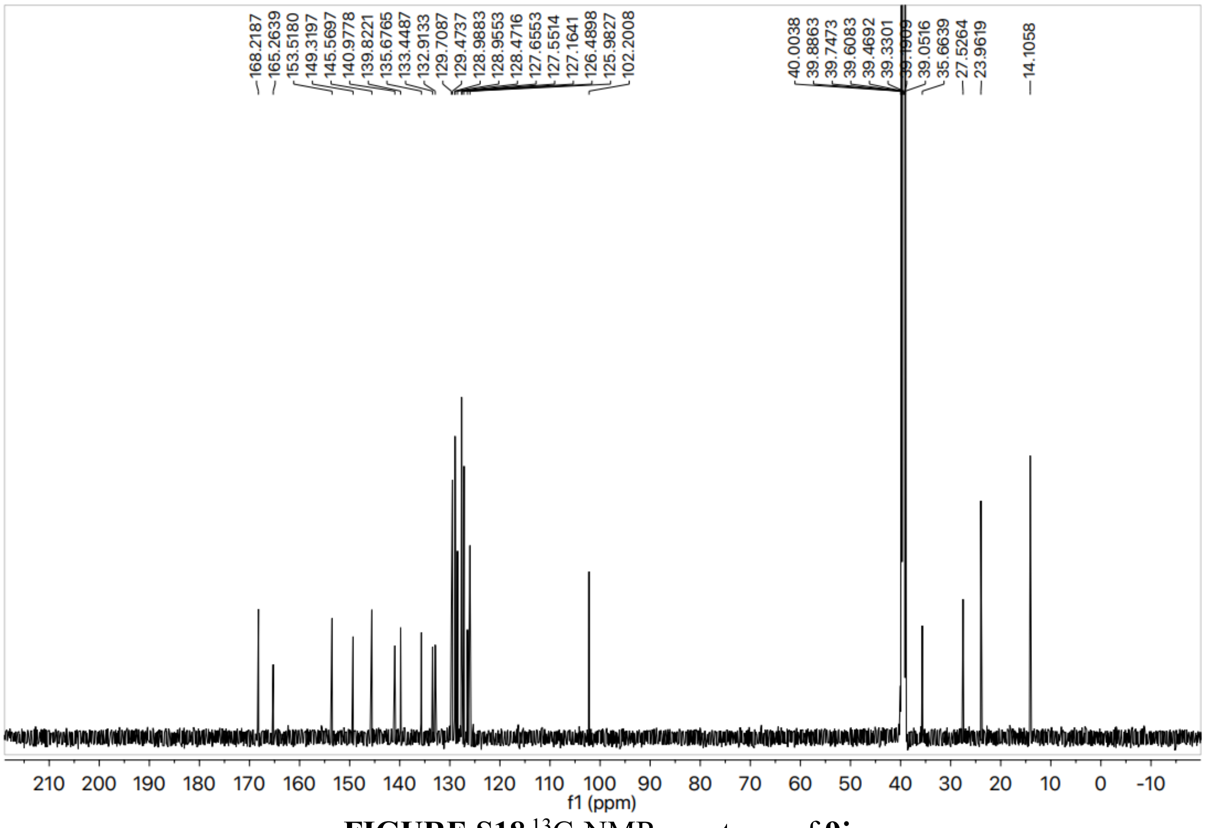

FIGURE S18 ^{13}C-NMR spectrum of **9j**

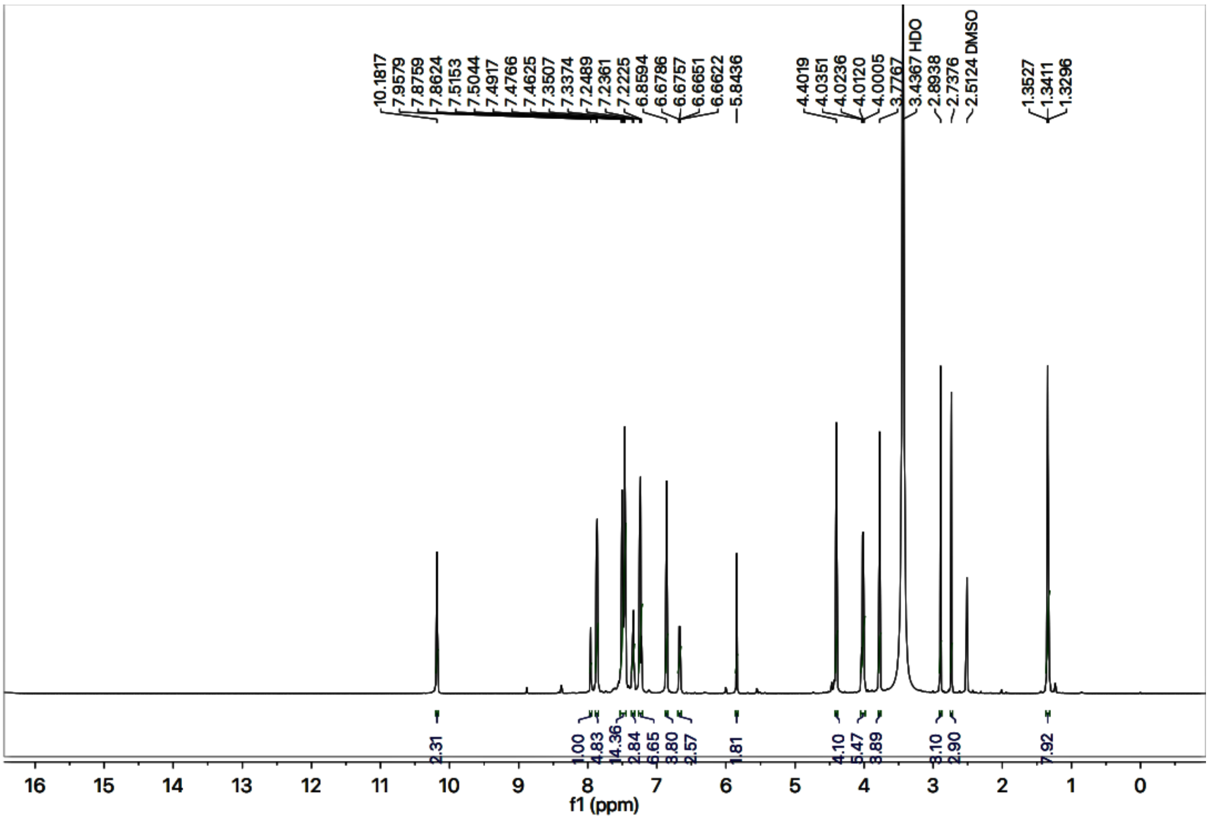

FIGURE S19 ^{1}H-NMR spectrum of **9k**

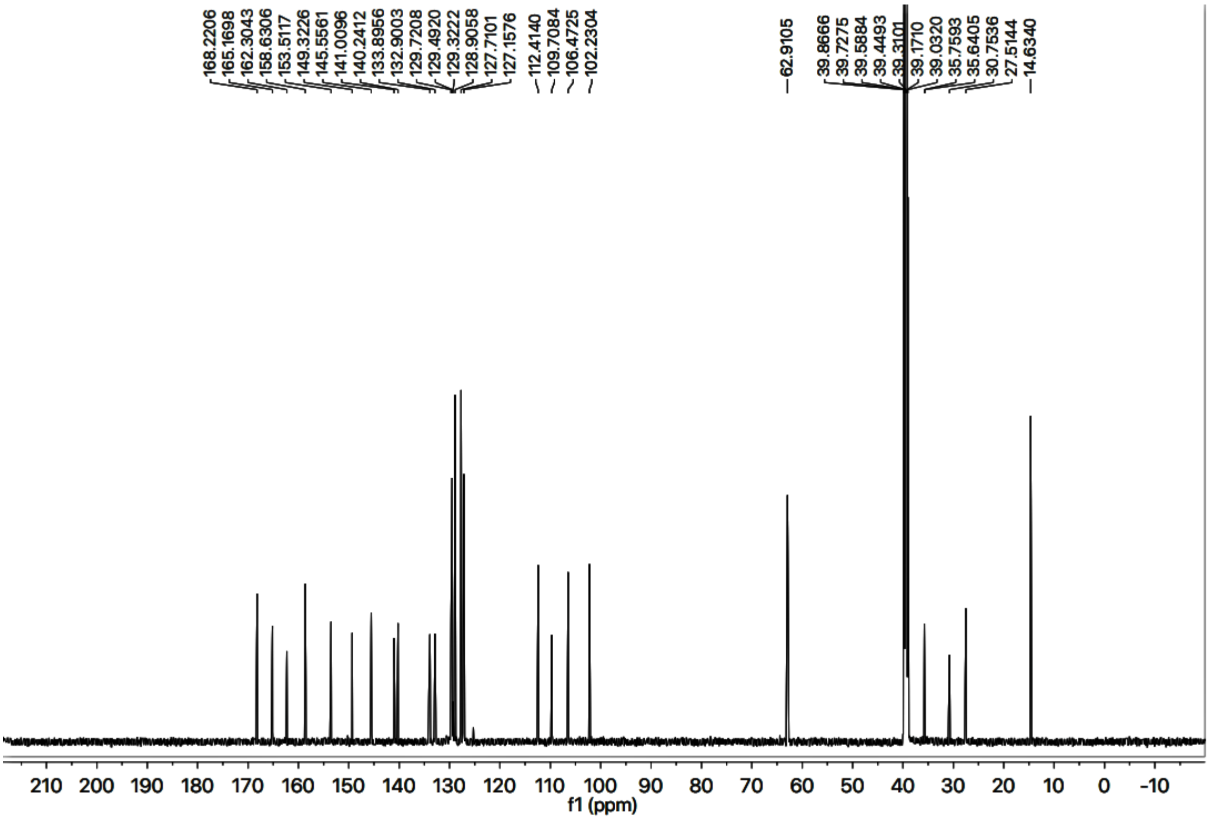

FIGURE S20 ^{13}C-NMR spectrum of **9k**

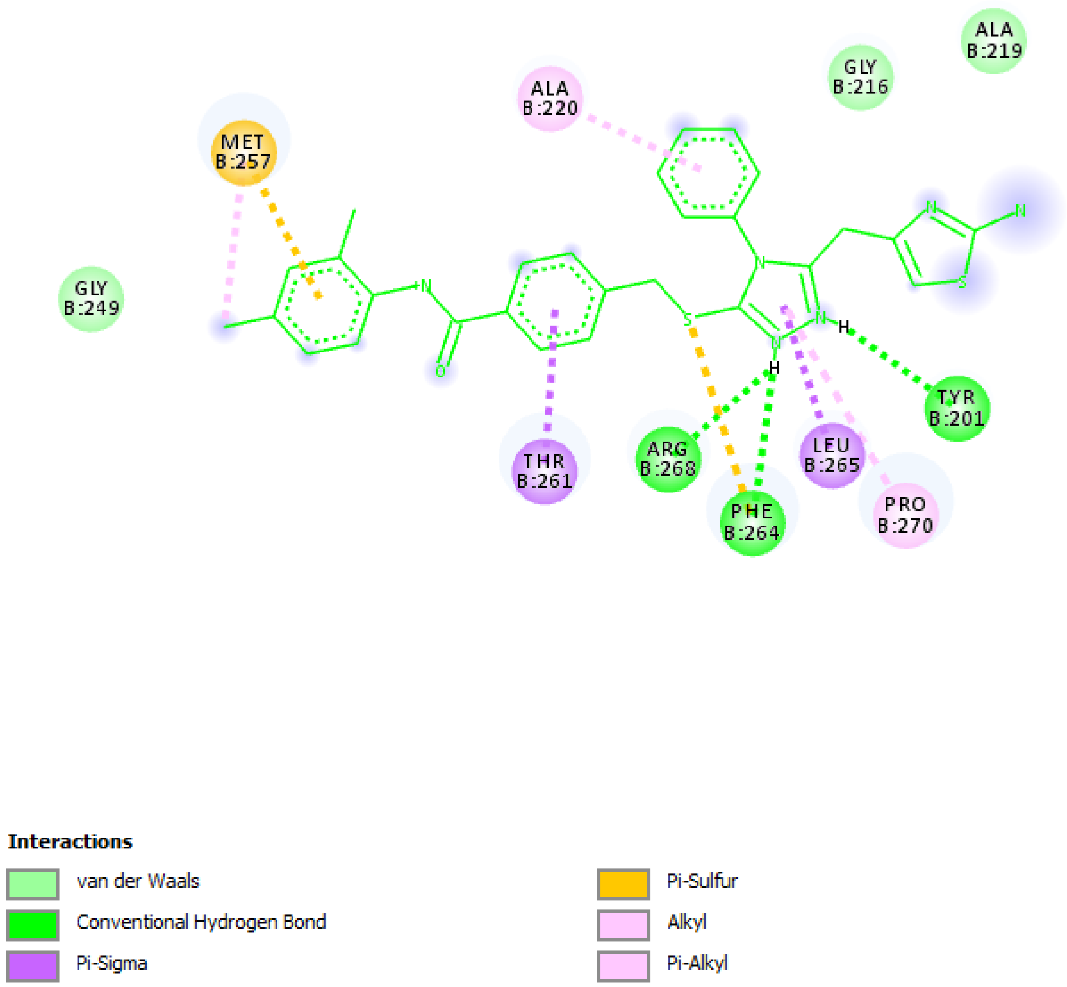

FIGURE S21 Docked configuration of **9a**

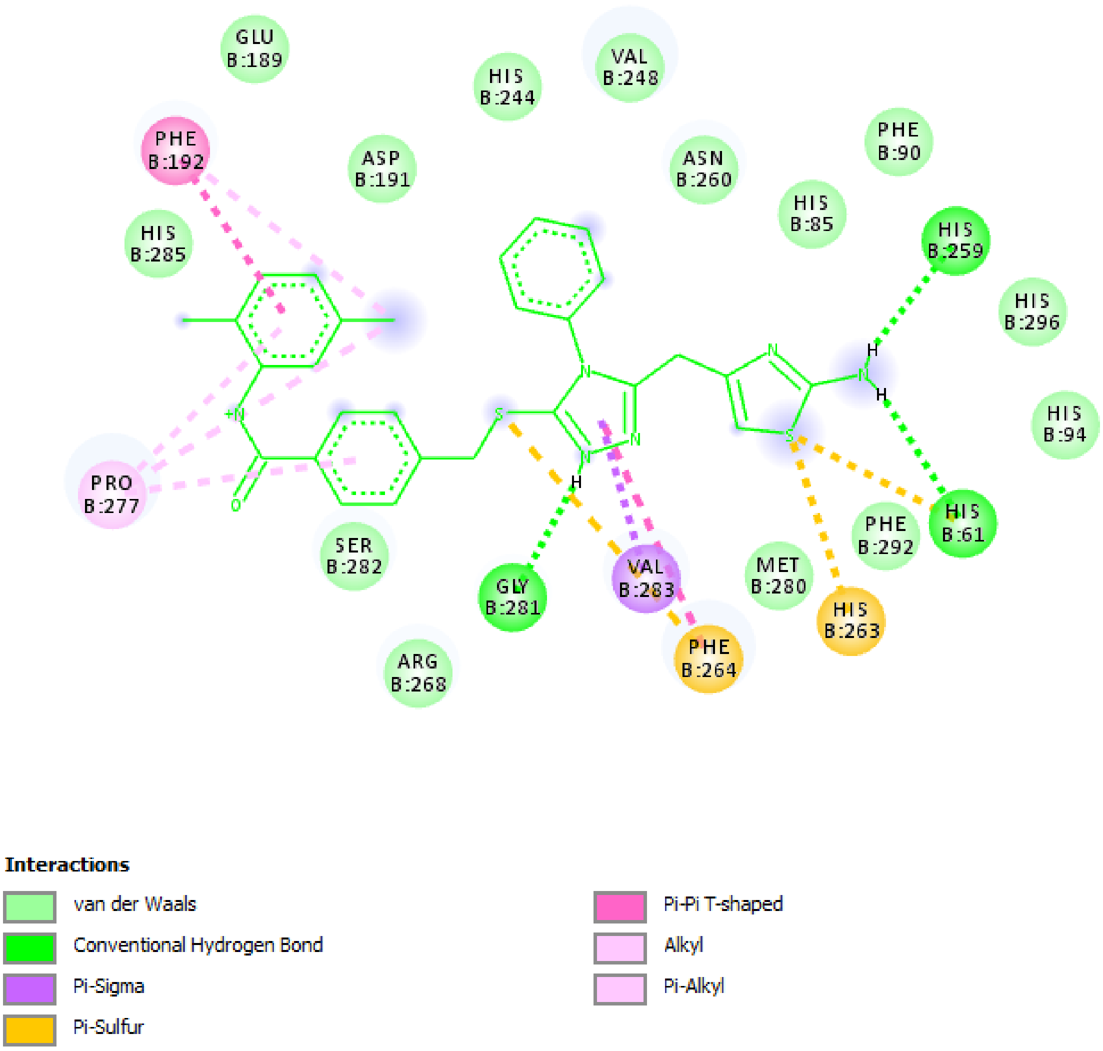

FIGURE S22 Docked configuration of **9b**

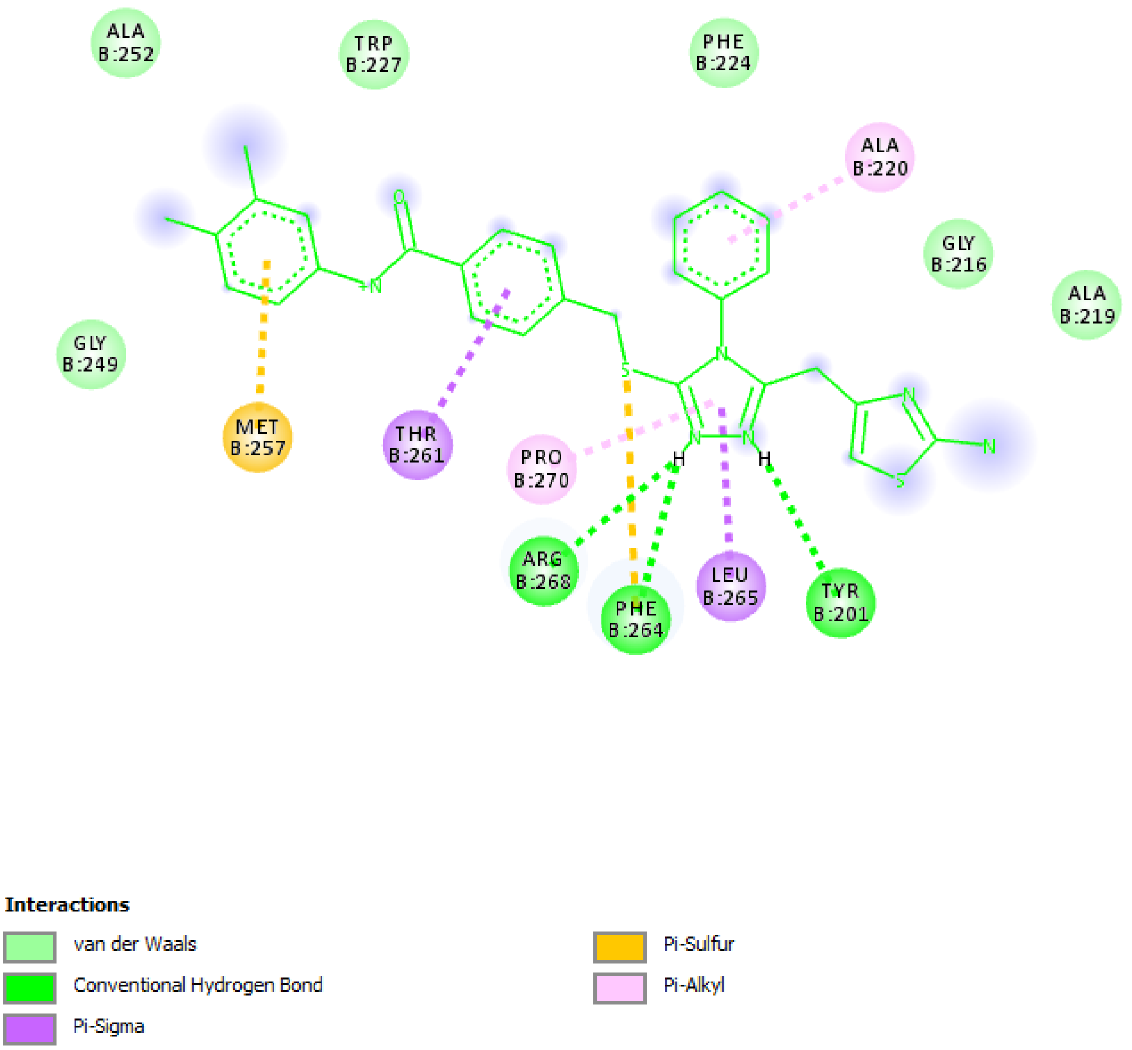

FIGURE S23 Docked configuration of **9d**

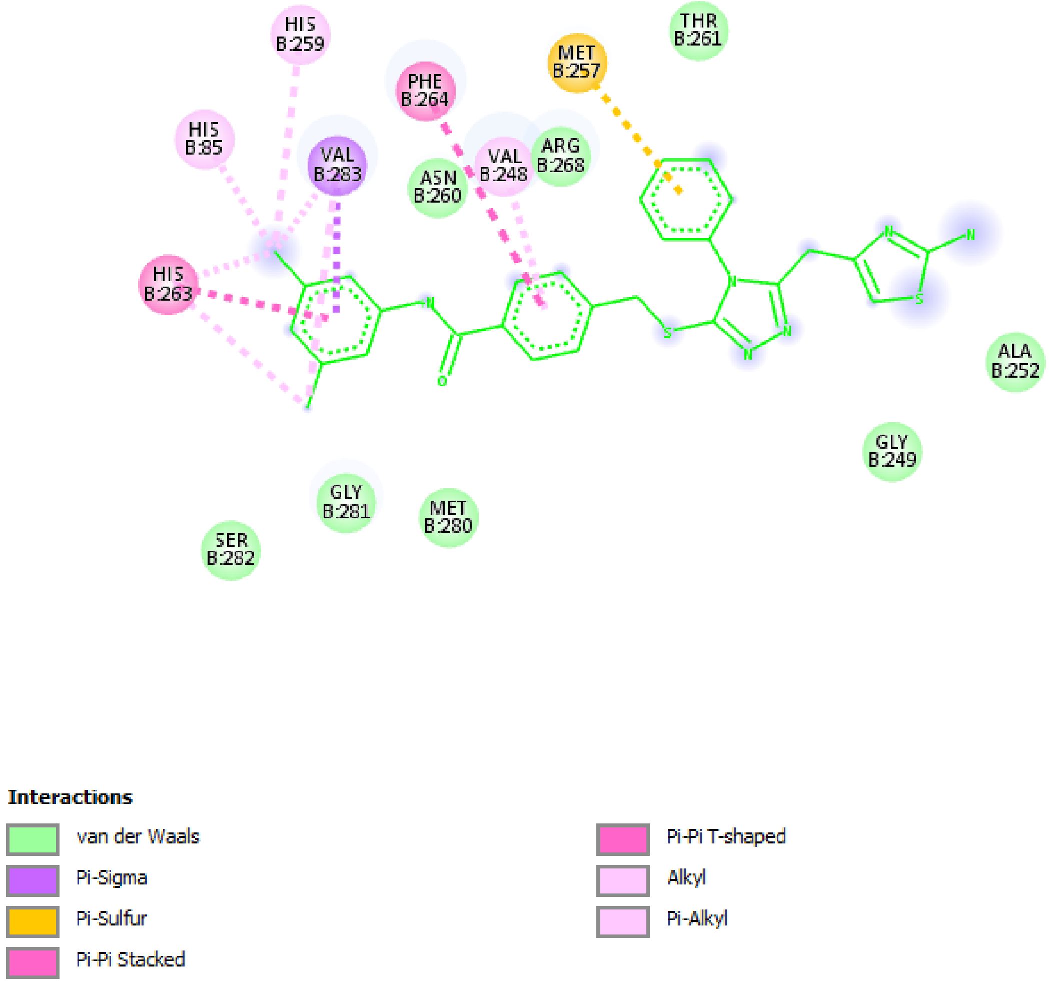

FIGURE S24 Docked configuration of **9e**

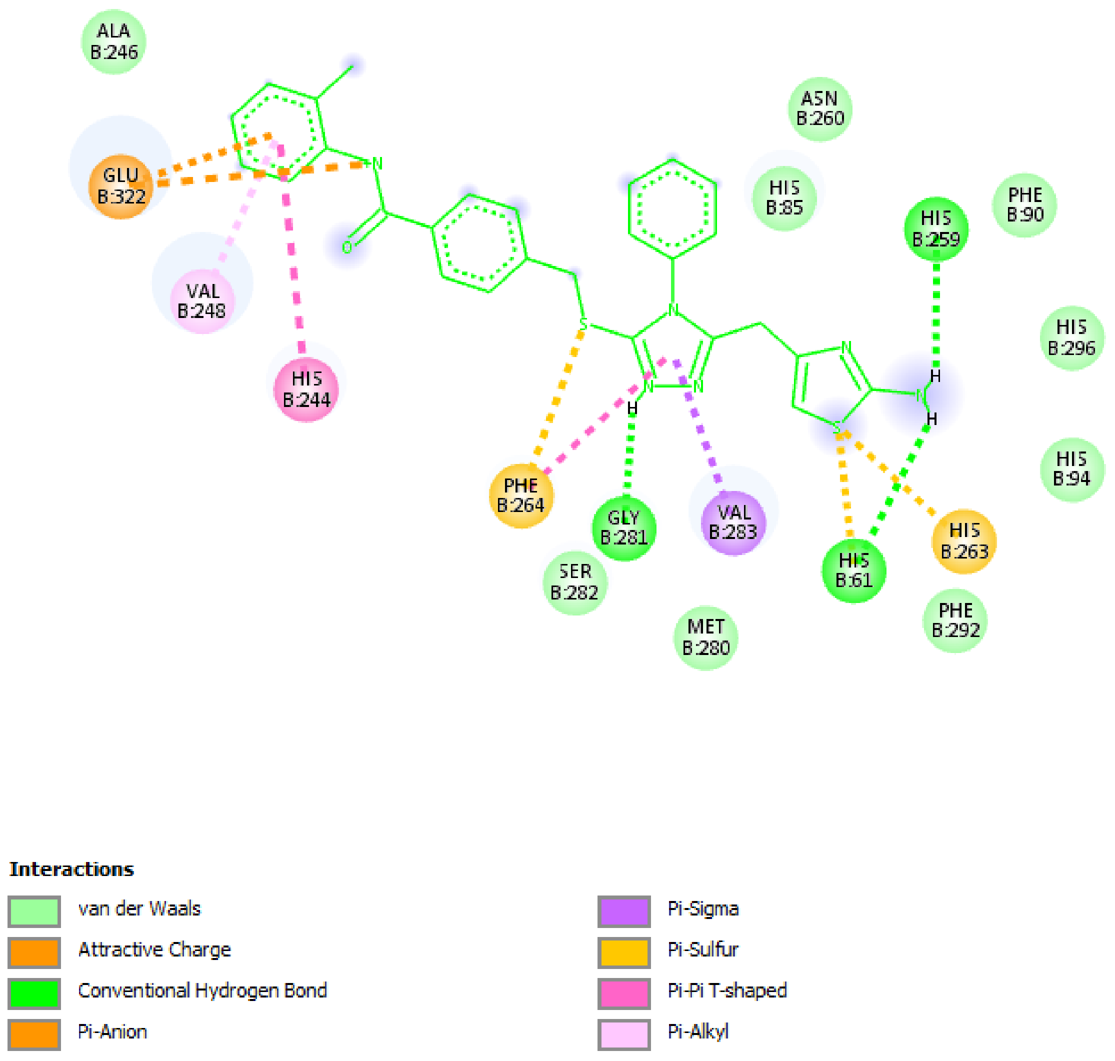

FIGURE S25 Docked configuration of **9f**

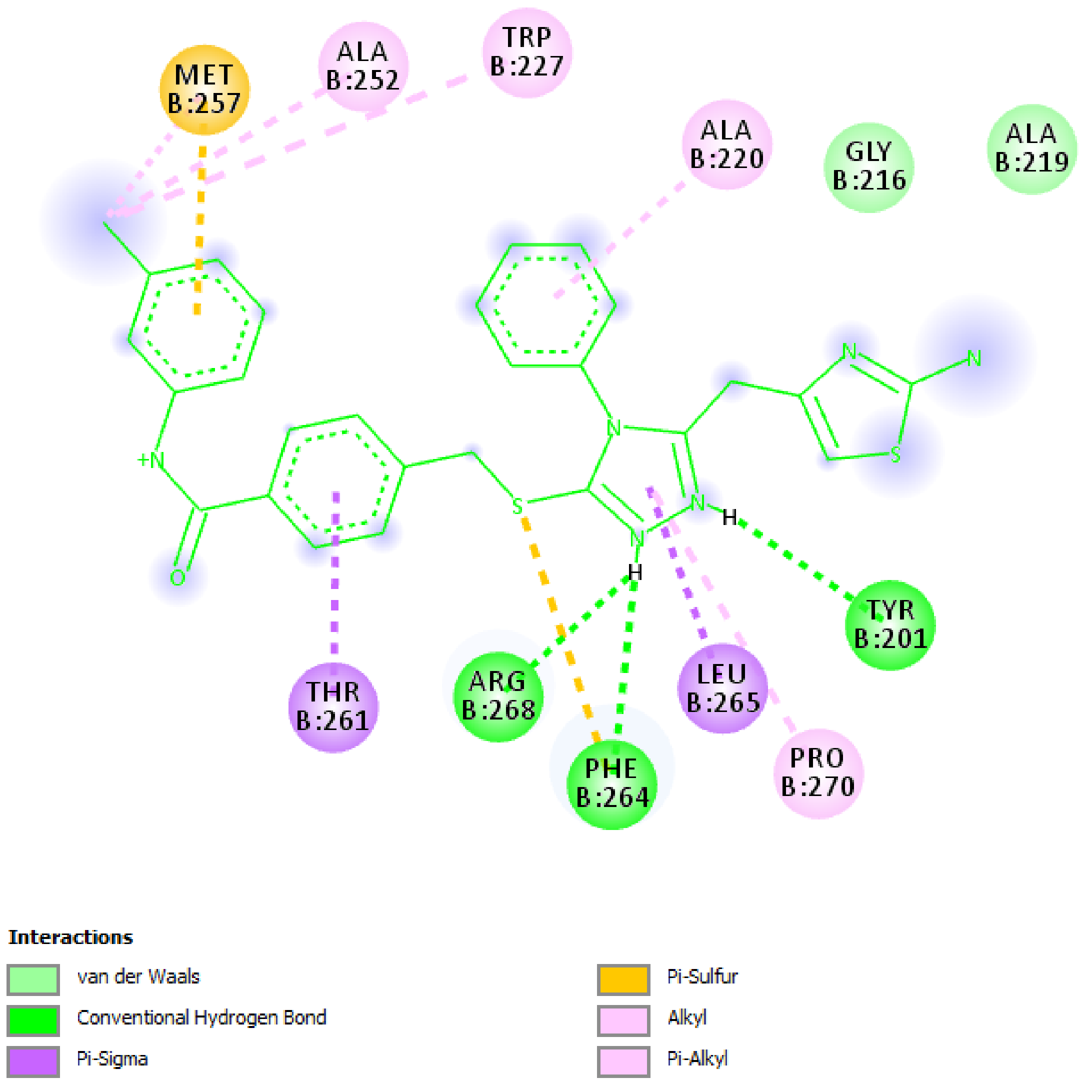

FIGURE S26 Docked configuration of **9g**

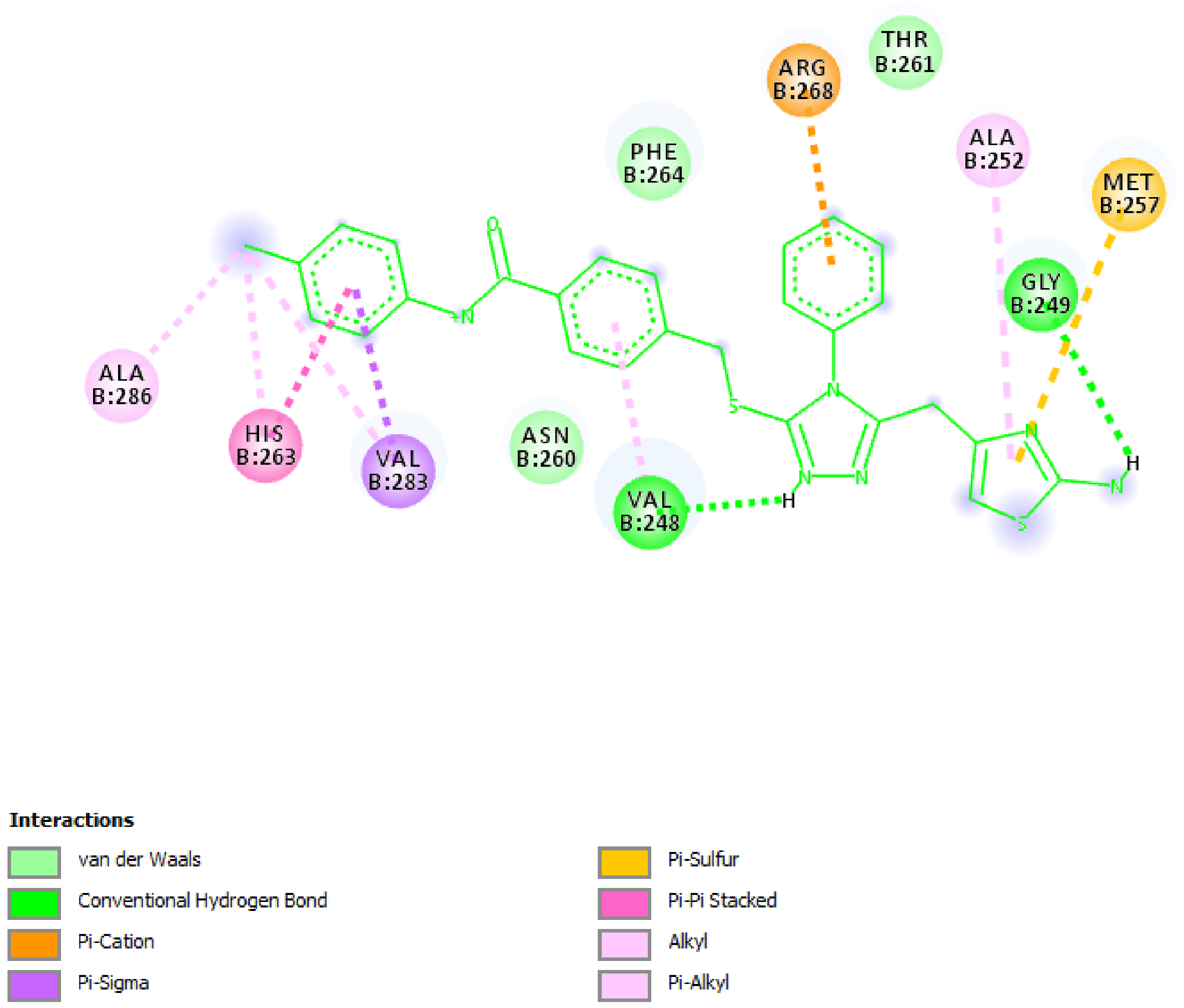

FIGURE S27 Docked configuration of **9h**

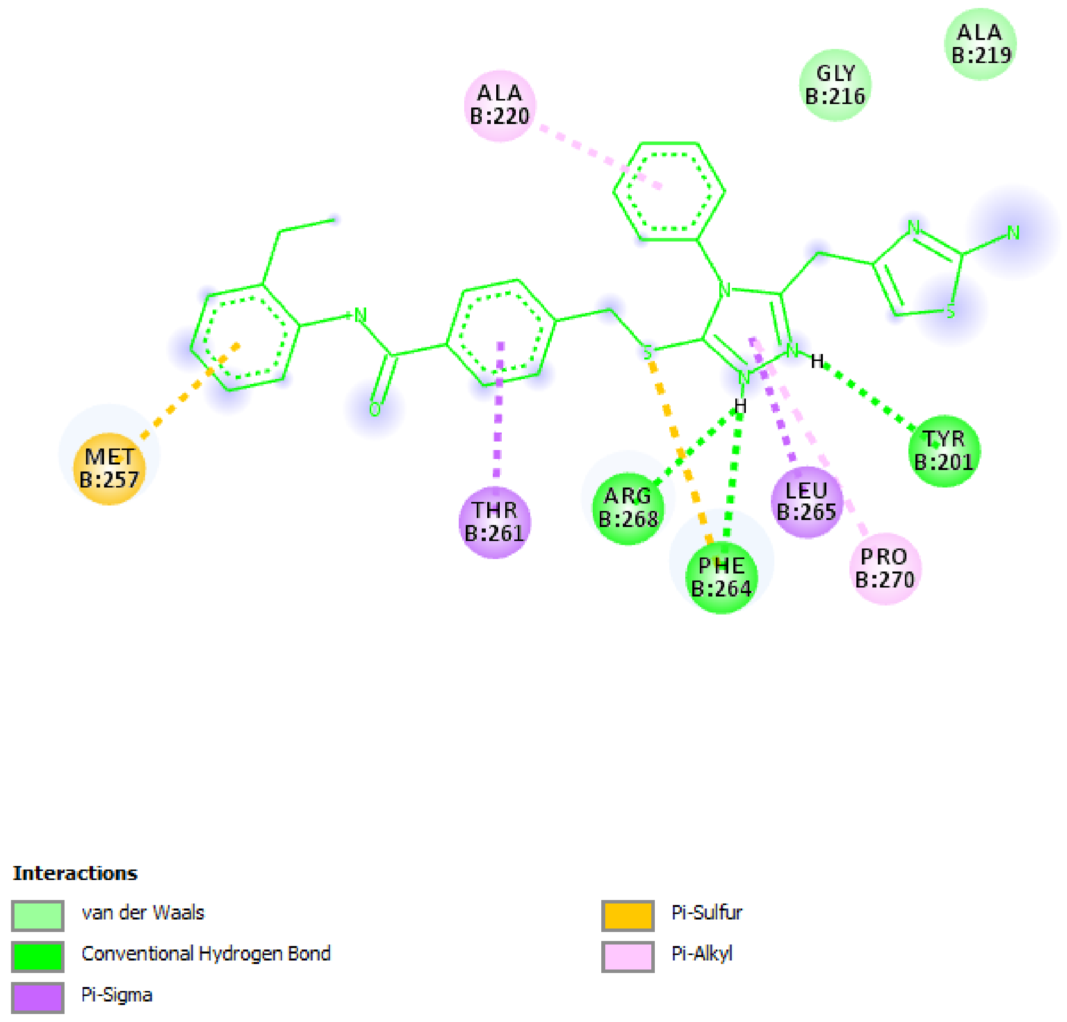

FIGURE S28 Docked configuration of **9i**

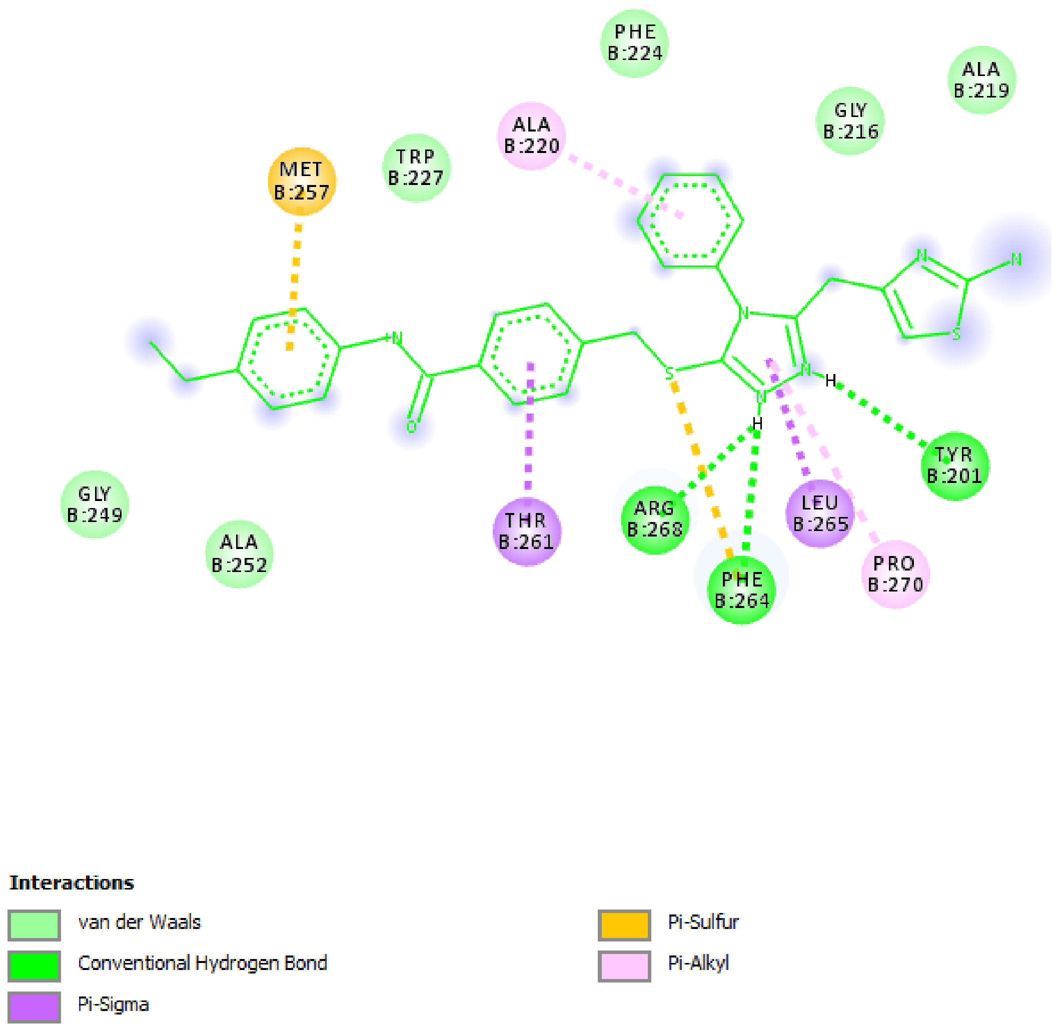

FIGURE S29 Docked configuration of **9j**

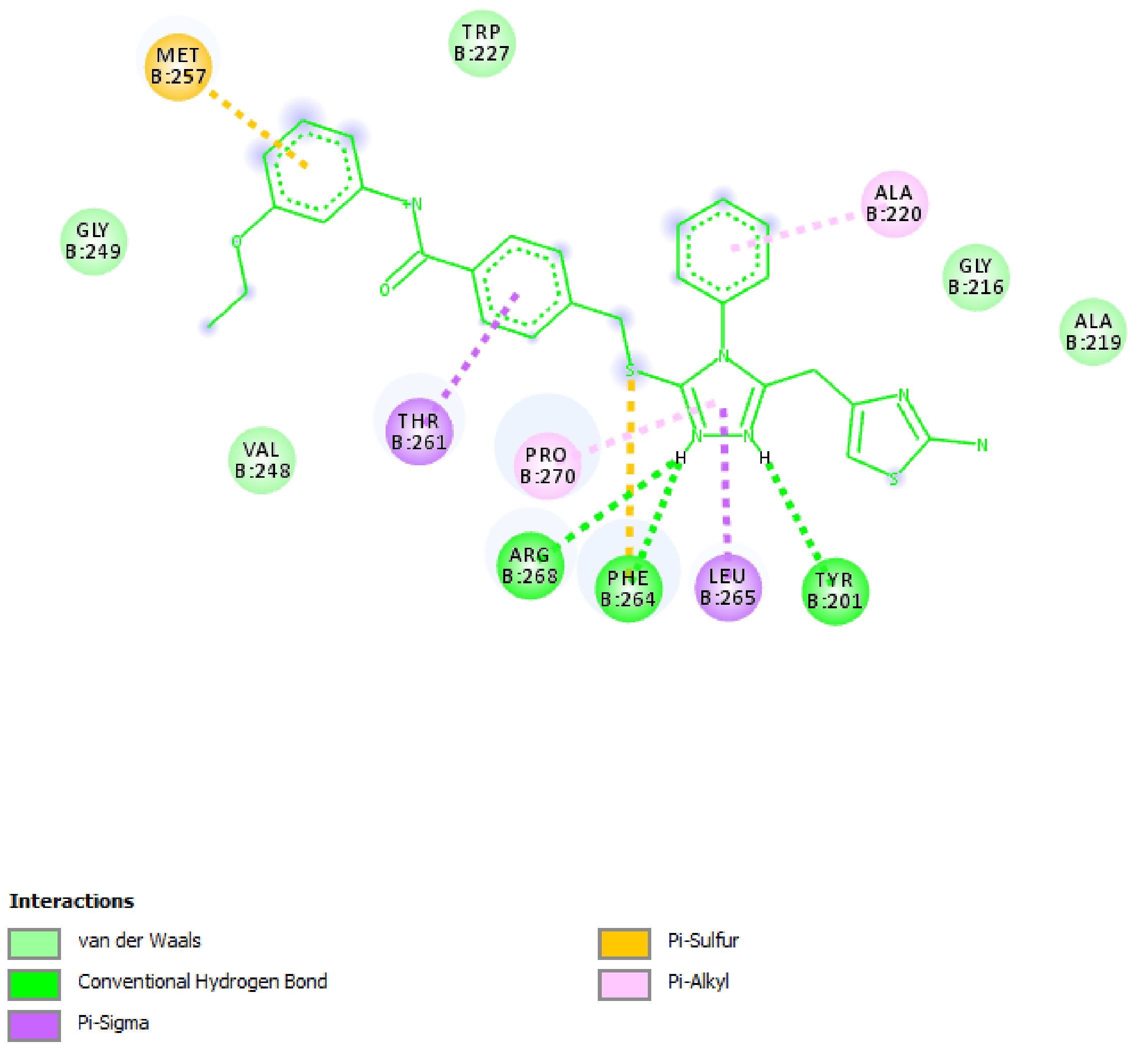

FIGURE S30 Docked configuration of **9k**
